# ZApprO versus ZÄPrO: Ergebnisse einer ersten Vergleichsstudie

**DOI:** 10.1007/s00103-023-03788-z

**Published:** 2023-10-20

**Authors:** Deniz Uzun, Theresa de Sousa, Steffani Görl, Silvia Brandt, Maria Giraki, Hari Petsos, Thorsten Blauhut, Stefan Heitkamp, Amira Begic, Karina Obreja, Babak Sayahpour, Sarah Bühling, Nicolas Plein, Andreas Möltner, Susanne Gerhardt-Szép, Tuğba Zahn

**Affiliations:** 1 Poliklinik für Zahnerhaltung, Zentrum der Zahn‑, Mund- und Kieferheilkunde (ZZMK, Carolinum), Goethe-Universität Frankfurt, Theodor-Stern-Kai 7 (Haus 29), 60596 Frankfurt am Main, Deutschland; 2 Poliklinik für Zahnärztliche Prothetik, Zentrum der Zahn‑, Mund- und Kieferheilkunde (ZZMK, Carolinum), Goethe-Universität Frankfurt, Frankfurt am Main, Deutschland; 3 Poliklinik für Parodontologie, Zentrum der Zahn‑, Mund- und Kieferheilkunde (ZZMK, Carolinum), Goethe-Universität Frankfurt, Frankfurt am Main, Deutschland; 4 Poliklinik für Zahnärztliche Chirurgie und Implantologie, Zentrum der Zahn‑, Mund- und Kieferheilkunde (ZZMK, Carolinum), Goethe-Universität Frankfurt, Frankfurt am Main, Deutschland; 5 Poliklinik für Kieferorthopädie, Zentrum der Zahn‑, Mund- und Kieferheilkunde (ZZMK, Carolinum), Goethe-Universität Frankfurt, Frankfurt am Main, Deutschland; 6 Qualitätsmanagement Lehre, Qualitätssicherung Prüfungen, Medizinische Fakultät Heidelberg, Heidelberg, Deutschland

**Keywords:** Zahnmedizin, Ausbildung, Fragebogen, Evaluation, Curriculum, Dentistry, Education, Questionnaire, Evaluation, Curriculum

## Abstract

**Hintergrund:**

Mehr als 60 Jahre nach dem Erlass der ersten Approbationsordnung für Zahnärzte (ZÄPrO) trat im Jahr 2020 eine neue Approbationsordnung für Zahnärzte und Zahnärztinnen (ZApprO) in Kraft. Ziele dieser Untersuchung waren die Evaluation und ein Vergleich der auf den unterschiedlichen gesetzlichen Grundlagen basierenden Lehrveranstaltungen „Kurs der Technischen Propädeutik“ (TPK) und „Zahnmedizinische Propädeutik mit Schwerpunkt Dentale Technologie“ (ZPDT).

**Methoden:**

Nach Abschluss der Veranstaltungen wurden folgende Parameter untersucht: (1.) theoretisches und praktisches Wissen, (2.) reguläre fachbereichsinterne Evaluation durch die Lernenden, (3.) spezielle Evaluation der Lernbedingungen aus Sicht der Lernenden und (4.) aus Sicht der Lehrenden. Die theoretischen und praktischen Prüfungen und die Fragebögen wurden hinsichtlich ihrer teststatistischen Kenngrößen (Schwierigkeit, Trennschärfe, interne Konsistenz) analysiert. Gruppenvergleiche zwischen TPK und ZPDT erfolgten durch t‑Tests für unabhängige Gruppen.

**Ergebnisse:**

Lediglich bei der Evaluation zur Erfassung der speziellen Lernbedingungen aus Sicht der Lernenden konnten signifikante Unterschiede festgestellt werden, wobei die theoretische und praktische Wissensvermittlung im TPK niedriger als im ZPDT bewertet wurde.

**Diskussion:**

Die vergleichbaren Ergebnisse der Wissensüberprüfungen und der regulären Evaluation, trianguliert mit der umfangreichen Evaluation durch Lernende und Lehrende, ermöglichten eine umfassende Beurteilung beider Veranstaltungen. Die ermittelten signifikanten Unterschiede eröffnen Möglichkeiten zur Optimierung des neu implementierten ZPDT-Kurses.

**Zusatzmaterial online:**

Zusätzliche Informationen sind in der Online-Version dieses Artikels (10.1007/s00103-023-03788-z) enthalten.

## Abstract (en)

**Background:**

More than 60 years after the enactment of the first Licensing Regulations for Dentists (ZÄPrO), a new Licensing Regulations for Dentists, the ZApprO, was implemented in 2020. The aims of this study were to evaluate and compare the “Course in Technical Propaedeutics” (TPK) with the course “Dental Propaedeutics with a Focus on Dental Technology” (ZPDT), which are based on different legal foundations.

**Methods:**

The following parameters were compared after completion of the courses: (1) theoretical and practical knowledge, (2) regular department-internal learner evaluation, and (3) special evaluation of learning conditions by the learners and (4) by the instructors. The theoretical and practical exams and the questionnaires were analyzed in terms of their test-statistical characteristics (difficulty, discrimination index, internal consistency). Group comparisons between TPK and ZPDT were conducted using t‑tests for independent groups.

**Results:**

Significant differences were only detected in the students’ evaluation of their learning conditions, with theoretical and practical knowledge transfer being rated lower in the TPK than in the ZPDT.

**Discussion:**

The comparable results of the knowledge assessments and the regular learner evaluation, triangulated with the comprehensive learner and instructor evaluation, allowed a broad assessment of both courses. The significant differences identified provide opportunities for optimization of the newly implemented ZPDT course.

## Hintergrund

Die von der Deutschen Gesellschaft für Zahn‑, Mund- und Kieferheilkunde (DGZMK) und den zahnärztlichen Körperschaften vorangetriebene Neubeschreibung der Zahnheilkunde mit einer stärkeren Hinwendung zur Prävention innerhalb der vergangenen 20 Jahre ist in der zahnärztlichen Praxis längst Realität geworden. Mehr als 60 Jahre nach Erlass der ersten Prüfungs- beziehungsweise Approbationsordnung für Zahnärzte (ZÄPrO) im Jahr 1955 folgte der Verordnungsgeber am 01.10.2020 den Forderungen nach einer zeitgemäßen, am heutigen Stand der zahnmedizinischen Wissenschaft orientierten Ausbildungsordnung [1, 2].

Die aktuelle Approbationsordnung für Zahnärzte und Zahnärztinnen (ZApprO) beinhaltet einige neue Schwerpunkte, die vor allem mit dem o. g. Präventionsgedanken, der steigenden Notwendigkeit von Interdisziplinarität und der zunehmenden Technisierung bzw. Digitalisierung in der Zahnmedizin zu erklären sind [3, 4]. Diese gilt es, prozedural möglichst breitflächig umzusetzen und das Erreichen der Ziele „regelmäßig und systematisch“ zu bewerten [1].

Die Evaluation von Curricula, deren Änderungen und direkten bzw. indirekten Auswirkungen wurden häufig in der Literatur thematisiert [4–16]. So gab es im Falle der ZApprO die Ansichten, dass der grundlegende Unterschied zwischen bisheriger und aktueller Approbationsordnung in der Neuorientierung an zahnärztlichen Behandlungsschritten, nicht aber in einer „Aufgabe zahnmedizinischer Studieninhalte“ liege. Ziel sei eine frühe, fachspezifische Kompetenzentwicklung bereits ab Studienbeginn [4]. Letzteres wurde auch durch die Entwicklung des Nationalen Kompetenzbasierten Lernzielkataloges Zahnmedizin (NKLZ) seit 2015 unterstützt [3, 16, 17].

Gleichzeitig wurden Bedenken an der ZApprO laut, insbesondere bezüglich einer vermuteten Reduzierung von Studieninhalten speziell im Bereich der Anfertigung von Zahnersatz, wobei als mögliche Vor-Ort-Lösung der Hinweis auf die jeweiligen Studienordnungen der einzelnen Universitäten deklariert wurde [2, 4].

Den Autorinnen und Autoren dieses Beitrags sind zum jetzigen Zeitpunkt keine Vergleichsuntersuchungen zwischen bisherigen und aktuellen sogenannten Propädeutikkursen im ersten Abschnitt des Zahnmedizinstudiums bekannt. Genau diese Lücke soll die vorliegende Studie mit dem Vergleich der Lehrveranstaltungen „Kurs der Technischen Propädeutik“ (TPK) nach der alten und der Lehrveranstaltung „Zahnmedizinische Propädeutik mit Schwerpunkt Dentale Technologie“ (ZPDT) nach der neuen Approbationsordnung schließen.

Folgende Parameter wurden verglichen:

1. theoretische und praktische Prüfungsergebnisse,
2. fachbereichsinterne Evaluation zur Erfassung genereller Rahmenbedingungen aus Sicht der Lernenden,
3. Evaluation zur Erfassung der speziellen Lehr- und Lernbedingungen aus Sicht der Lernenden mit Bezug zu spezifischen Themen wie Digitalisierung, Interdisziplinarität sowie Anatomie-Einführungskursen, die im ZPDT erstmals unmittelbar vor der Lehrveranstaltung stattfanden,
4. Evaluation aus Sicht der Lehrenden.

Hauptfragestellung der vorliegenden Untersuchung war somit, ob sich Unterschiede in der Bewertung der beiden Lehrveranstaltungen bezüglich der oben genannten Parameter feststellen lassen.

Die gewonnenen Erkenntnisse sollen dazu dienen, die Lehrveranstaltung ZPDT gegebenenfalls gezielt zu optimieren.

## Methoden

Die Datenerhebung fand von Juli bis Dezember 2022 im Zentrum für Zahn‑, Mund- und Kieferheilkunde (Carolinum) der Goethe-Universität Frankfurt am Main statt. An der Lehrveranstaltung TPK nahmen 39 Lernende (26 weiblich, 13 männlich) mit einem Durchschnittsalter von 24,10 (±4,24) Jahren teil, die überwiegend dem 5. und 6. Fachsemester (ZÄPrO) zuzuordnen waren. Sie befanden sich somit nicht mehr in der Regelstudienzeit. Der ZPDT-Kurs bestand aus 45 Lernenden (32 weiblich, 13 männlich) mit einem Durchschnittsalter von 19,96 (±1,17) Jahren, welche das Studium gerade begonnen hatten und sich somit im 1. Fachsemester befanden (ZApprO). Die Ein- und Ausschlusskriterien sind der Tab. 1 im Onlinematerial zu entnehmen.

### Allgemeiner Aufbau sowie Besonderheiten der Lehrveranstaltungen

Beide Veranstaltungen (Tab. 1) beinhalteten jeweils eine Vorlesung sowie ein Praktikum und schlossen mit einer summativen Prüfung ab, welche aus einem theoretischen (schriftlichen) und einem praktischen Teil bestand [18, 19]. Alle den Prüfungen zugrunde liegenden Items wurden an vorher definierten NKLZ-Lernzielen orientierend konzipiert. Voraussetzung für die Prüfungszulassung war, alle praktischen, in einem Testatheft nachzuweisenden Arbeitsschritte absolviert zu haben. Bei allen Prüfungen war eine einmalige Wiederholung möglich.

**Table 1:** 

		TPK	ZPDT
1	Rechtsgrundlage	ZÄPrO, Ausfertigungsdatum: 26.01.1955, zuletzt geändert durch Art. 34 des Gesetzes vom 06.12.2011 I 2515	ZApprO, Ausfertigungsdatum: 08.07.2019, zuletzt geändert durch Art. 1 der Verordnung vom 22.09.2021 I 4335
2	Studienordnung	Für den Studiengang Zahnmedizin der Goethe-Universität Frankfurt vom 03.09.2015 in der Fassung vom 02.05.2019	Für den Studiengang Zahnmedizin der Goethe-Universität Frankfurt vom 06.05.2021
3	Zeitraum (Anzahl der Wochen)	Anfang August–Mitte September 2022 (7 SW)	Mitte April–Mitte Juli 2022 (14 SW)
4	SWS (Vorlesung + Praktikum)	40 SWS^a,b^	2 SWS + 3 SWS = 5 SWS
5	SWS gesamt (3. × 4.)	7 SW × 40 SWS = 280 SS	14 SW × 5 SWS = 70 SS
6	Selbststudienzeiten (Vorlesung/Praktikum)	Vorlesung: 0 SS/Praktikum: 0 SS	Vorlesung: 51 SS/Praktikum: 76 SS
7	Gesamtkurszeit (5. + 6.)	280 SS	197 SS
8	Betreuungsrelation	1:20 (1 Lehrender auf 20 Lernende)	1:15 (1 Lehrender auf 15 Lernende)
9	Testatheft	32 Testate	32 Testate
10	Inhalte theoretischer Wissensvermittlung in didaktisch chronologischer Reihenfolge	Orale Strukturen	Orale Strukturen
		Grundlagen der Okklusion	Grundlagen der Okklusion
		Hygiene und Arbeitssicherheit	Hygiene und Arbeitssicherheit
		Unterkieferbewegungen und Artikulatoren	Unterkieferbewegungen und Artikulatoren
		Situationsabformung und Modellherstellung (konventionell und digital)	Situationsabformung und Modellherstellung (konventionell und digital)
		Präparationen	Präparationen
		Werkstoffkunde	Werkstoffkunde
		Arten von Zahnersatz und Qualitätskriterien	Arten von Zahnersatz und Qualitätskriterien
			Dentale Technologie in der Kieferorthopädie
			Parodontale Röntgendiagnostik
			Implantologie
			Implantologie aus parodontologischer Sicht
			Funktion und Ästhetik
11	Inhalte praktischer Wissensvermittlung in didaktisch chronologischer Reihenfolge	Knetübungen	Knetübungen
		Gipsübungen	Gipsübungen
		Aufwachsübungen	Aufwachsübungen
		Situationsabformungen und Modellherstellung	Situationsabformungen und Modellherstellung
		Übertragungsbogen, Einbau der Modelle und Splitcast-Kontrolle	Übertragungsbogen, Einbau der Modelle und Splitcast-Kontrolle
		Präparationen	Präparationen
		Digitale Abformung und Design der Restauration	Digitale Abformung und Design der Restauration
12	Creditpoints (Vorlesung/Praktikum)	Vorlesung: –/Praktikum: –	Vorlesung: 3/Praktikum: 4
13	NKLZ -basiert	Nein	Ja
14	Interdisziplinarität (beteiligte Polikliniken)	Nein (1 Poliklinik – Prothetik)	Ja (5 Polikliniken – Prothetik, Kons, PA, KFO, Chirurgie)

*ZÄPrO* Approbationsordnung für Zahnärzte, *ZApprO* Approbationsordnung für Zahnärzte und Zahnärztinnen, *SW* Semesterwochen, *SWS* Semesterwochenstunden, *SS* Semesterstunden (45 min), *NKLZ* Nationaler Kompetenzbasierter Lernzielkatalog Zahnmedizin, *Kons* Zahnerhaltung, *PA* Parodontologie, *KFO* Kieferorthopädie

^a^Aufteilung der SWS in Vorlesung und Praktikum curricular nicht definiert

^b^Aufgrund der Halbierung der SW von 14 auf 7 ergaben sich 2 × 20 curricular vorgegebene SWS

Die Lehrveranstaltung TPK basierte auf der alten ZÄPrO und fand innerhalb von 7 Semesterwochen mit jeweils 40 Semesterwochenstunden (SWS) von Anfang August bis Mitte September 2022 statt. Somit ergab sich eine Gesamtzeit von 280 Semesterstunden [18].

In der neuen Studienordnung sind der modulare Aufbau und die Ausweisung der Lehrveranstaltungen nach Maßstäben des europäischen Credit-Transfer-Systems vorgegeben [19]. Die Lehrveranstaltung ZPDT wurde interdisziplinär und kompetenzorientiert (NKLZ-basiert) erstellt. Hierzu gehörte die Etablierung einer Curriculums-Arbeitsgruppe (CA), die aus 8 Mitgliedern aus den Bereichen Prothetik (2), Zahnerhaltung, inkl. Parodontologie (2), Oralchirurgie (1), Kieferorthopädie (1) sowie Studierenden der Fachgruppe Zahnmedizin (2) bestand.

Erstellt wurde die Lehrveranstaltung während insgesamt 9 Sitzungen der CA, die von August bis Dezember 2021 stattfanden. Zusätzlich wurde erstmalig ein Anatomie-Einführungskurs zu Beginn angeboten. Im TPK hingegen wurde der Anatomiekurs schon über 3 Semester gelehrt.

Die Lehrveranstaltung fand innerhalb von 14 Semesterwochen mit jeweils 5 SWS (2 SWS für die Vorlesung + 3 SWS für das Praktikum) von Mitte April bis Mitte Juli 2022 statt. Somit belief sich die Gesamtzeit der Semesterstunden in Präsenz auf 70.

Zusätzlich wurden Selbststudienzeiten definiert, welche für die Vorlesung (51) und das Praktikum (76) insgesamt 127 Semesterstunden umfassten. Dadurch betrug die Gesamtzeit der Lehrveranstaltung 197 Semesterstunden. Im Rahmen des Selbststudiums wurden praktische und theoretische Aufgaben erledigt, die seitens der Lehrenden mit einem kontinuierlichen Feedback unterstützt wurden.

### Evaluation der Wissensüberprüfungen (Parameter 1)

In beiden Kursen wurde eine schriftliche Prüfung im Einfach-Wahl-Verfahren (Single Choice) durchgeführt, wobei entweder 45 Fragen in 70 min (TPK) oder 30 Fragen in 45 min (ZPDT) zu beantworten waren. 14 der Klausurfragen waren in beiden Prüfungen identisch und bildeten die Grundlage der späteren Gegenüberstellung der erreichten Ergebnisse.

Den 14 gemeinsamen Klausurfragen wurde jeweils ein entsprechendes NKLZ-Lernziel zugeordnet. In Abhängigkeit von der Selbsteinschätzung der Lernenden hinsichtlich des entsprechenden Lernziels in der nachfolgenden Evaluation und den zugeordneten Resultaten in der Klausur (richtige oder falsche Antwort) war es somit möglich, Aussagen über die Übereinstimmung von Selbst- und Fremdeinschätzung (Bewertung durch Lehrende) zu treffen.

Die praktische Wissensüberprüfung beinhaltete in beiden Kursen die Präparation des Zahnes 11 im Phantomkopf zur Aufnahme einer vestibulär verblendeten Verblendmetallkrone innerhalb von 2 h. Diese bildete die Grundlage der späteren Gegenüberstellung der erreichten Ergebnisse. Zusätzlich musste in der TPK-Prüfung der Zahn 26 aus 250 g Plastilin-Knetmasse in Übergröße modelliert werden (Prüfungszeit 3 h), während in der ZPDT-Prüfung der gleiche Zahn im Labor aus Modellierwachs in natürlicher Größe modelliert werden sollte (Prüfungszeit 2,5 h).

**Figure 1:**
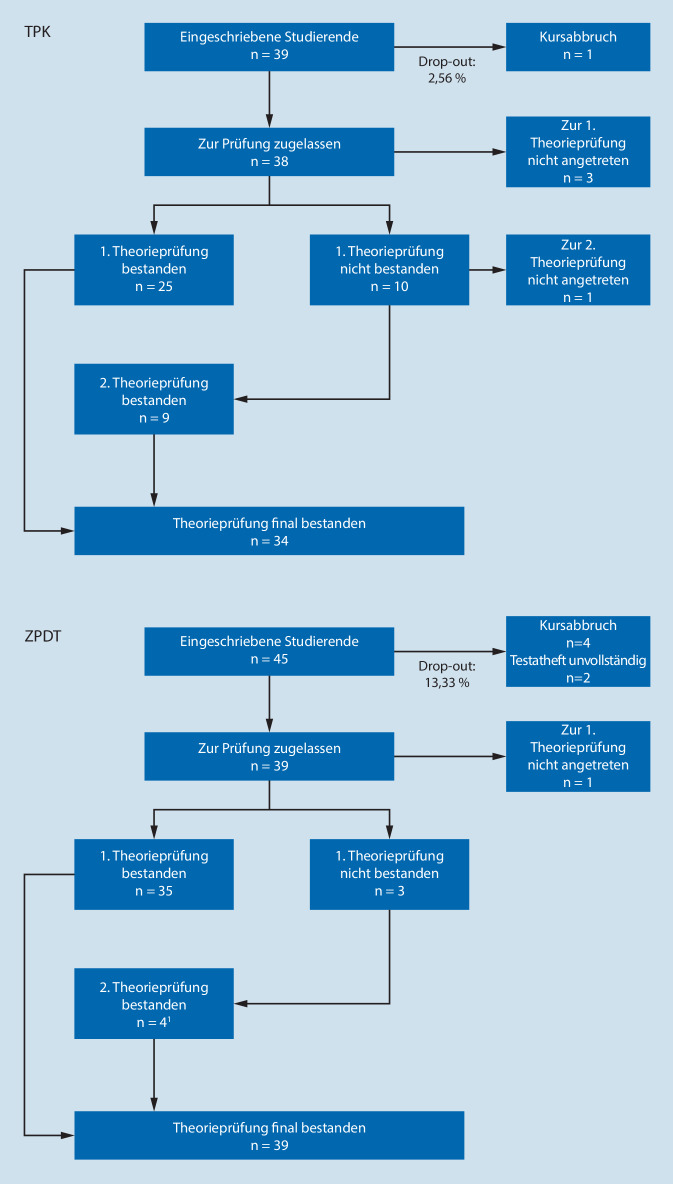

Bewertet wurden alle praktischen Prüfungen anhand objektiver Bewertungskriterien durch dieselben kalibrierten Prüfenden, bestehend aus Zahnärztinnen und -ärzten der Poliklinik für Zahnärztliche Prothetik (siehe Abb. 1–3 im Onlinematerial). Es war obligatorisch, beide Prüfungsteile erfolgreich zu absolvieren, um die praktische Prüfung insgesamt zu bestehen. Auch im Rahmen der praktischen Wissensüberprüfung konnten unter Bezugnahme auf die Selbsteinschätzung hinsichtlich entsprechender praktischer Lernziele Aussagen über Selbst- und Fremdeinschätzung getroffen werden.

### Fachbereichsinterne Evaluation zur Erfassung genereller Rahmenbedingungen aus Sicht der Lernenden (Parameter 2)

Die nach jeder Lehrveranstaltung regulär stattfindende, fachbereichsinterne Evaluation aus Sicht der Lernenden fand jeweils am Ende des ZPDT- und TPK-Kurses anonym statt. Sie bestand aus 4 Abschnitten:

A. Angaben zur Organisation,
B. Angaben zur Lehrveranstaltung,
C. Ergänzung digitale Lehre und
D. Angaben zur Person bzw. zum aktuellen Studiengang (siehe Tab. 2 im Onlinematerial).

### Fragebogen zur Erfassung der speziellen Lehr- und Lernbedingungen aus Sicht der Lernenden (Parameter 3)

Zur Erfassung der speziellen Lehr- und Lernbedingungen wurde ein mehrteiliges Evaluationsinstrument nach der Delphi-Methode erarbeitet [20, 21]. Basierend auf Fragebögen aus ähnlichen Publikationen wurde ein initiales Instrument entwickelt [22–30]. Dieses wurde anschließend sukzessive in 23 Delphi-Runden optimiert und inhaltlich an die beiden Lehrveranstaltungen angepasst. Der finale Fragebogen bestand aus 5 Abschnitten:

E. allgemeine Angaben zu Stammdaten für das Zahnmedizinstudium,
F. theoretische Wissensvermittlung (sowohl in der Vorlesung als auch im Praktikum),
G. praktische Vermittlung von Kompetenzen,
H. sonstige Angaben (u. a. Angaben zur Einschätzung der Relevanz eines Anatomiekurses),
I. subjektive Einschätzung der erzielten Kompetenzen anhand theoretischer und praktischer NKLZ-Lernziele (siehe Abb. 4–10 im Onlinematerial).

In einem Pretest wurde der Fragebogen an einer Referenzgruppe (5 über die Studie informierte Lernende aus den Fachsemestern 5–9, ZÄPrO) vorab online getestet. Das Meeting wurde mit der Zustimmung aller aufgezeichnet. Dieser Pretest wurde anhand der Think-aloud-Methode durchgeführt [31] und im Anschluss die finale Version des Fragebogens konstruiert.

Der Einsatz der Fragebögen zur Erfassung der speziellen Lehr- und Lernbedingungen erfolgte in beiden Kohorten nach Abschluss der theoretischen und praktischen Wiederholungsprüfungen digital (Software „Sosci-Survey“ 3.2.44, München). Die Befragung begann am 14.10.2022. Im Abstand von je 2 Wochen wurden die Lernenden in 3 Erinnerungs-E-Mails zur Teilnahme gebeten. Um die Rücklaufquote zu erhöhen, wurden die Teilnehmenden in der 48. und 51. Kalenderwoche im Rahmen von Lehrveranstaltungen nochmals direkt an die Bearbeitung der ausgeteilten Bögen erinnert, welche dann pseudonymisiert bearbeitet und anonym ausgewertet wurden.

### Fragebogen zur Erfassung der speziellen Lehr- und Lernbedingungen aus Sicht der Lehrenden (Parameter 4)

Um zusätzlich eine Evaluation aus Sicht der Lehrenden zu erhalten, wurde ein Fragebogen (angelehnt an den Fragebogen zur Erfassung spezieller Lehr- und Lernbedingungen der Lernenden) erstellt, welcher am Ende der jeweiligen Kurse anonym von den Personen bearbeitet wurde, die an diesen mitgewirkt hatten (siehe Abb. 11–12 im Onlinematerial).

Die statistische Auswertung fand in Zusammenarbeit mit der Abteilung „Qualitätssicherung Prüfungen“ der Medizinischen Fakultät Heidelberg statt. Nach einer explorativen Analyse der erhobenen Daten (Mittelwerte (MW), Standardabweichungen, Mediane, Minima, Maxima) erfolgten die Bestimmung teststatistischer Kennwerte sowie Gruppenvergleiche mittels Welch-t-Test für unabhängige Stichproben. Es wurden die Software SAS for Windows 9.3 (SAS Institute, Cary, NC, USA) und R 4.2.1 (The R Foundation for Statistical Computing, Wien, Austria) verwendet.

## Ergebnisse

Eine Übersicht der Ergebnisse befindet sich in Tab. 2.

**Table 2:** 

	TPK	ZPDT	Signifikanz^a^
*1. Parameter (Wissensüberprüfungen)*			
Theoretische Wissensüberprüfung (14 identische Klausurfragen)^b^	9,74 ± 2,12	9,97 ± 2,26	0,6538
Praktische Wissensüberprüfung (identische Präparationsprüfung)^c^	10,50 ± 2,93	10,84 ± 2,89	0,6150
*2. Parameter (fachbereichsinterne Evaluation)*^d^			
Insgesamt	2,68	2,49	k. A. m.^f^
Angaben zur Organisation	2,36	2,06	k. A. m.^f^
Angaben zur Lehrveranstaltung	2,98	2,73	k. A. m.^f^
Angaben zur digitalen Lehre	2,46	2,41	k. A. m.^f^
*3. Parameter (Evaluation spezieller Lehr- und Lernbedingungen der Lernenden)*^e^			
Insgesamt	2,30 ± 0,36	2,60 ± 0,42	0,0119*
Theoretische Wissensvermittlung	2,16 ± 0,38	2,52 ± 0,38	0,0015*
Praktische Wissensvermittlung	1,93 ± 0,51	3,09 ± 0,55	< 0,00001*
Sonstige Angaben	1,88 ± 0,41	2,04 ± 0,49	0,2191
Lernziele insgesamt	2,62 ± 0,48	2,51 ± 0,55	0,4654
*4. Parameter (Evaluation spezieller Lehr- und Lernbedingungen der Lehrenden)*^e^			
Insgesamt	2,62 ± 0,44	2,75 ± 0,62	0,6278
Theoretische Wissensvermittlung	2,67 ± 0,54	2,69 ± 0,39	0,9291
Praktische Wissensvermittlung	2,66 ± 0,25	3,04 ± 0,98	0,3159

*Statistische Signifikanz (*p* ≤ 0,05)

^a^Nach Welch-t-Test

^b^Durchschnittliche Anzahl richtig beantworteter Klausurfragen (maximal 14 möglich)

^c^Durchschnittliche Anzahl erreichter Punkte (maximal 18 möglich)

^d^1 = stimme voll und ganz zu–6 = stimme überhaupt nicht zu

^e^1 = sehr gut/stimme voll zu–5 = mangelhaft/stimme gar nicht zu

^f^Keine näheren Angaben aus den vorliegenden Daten des Fachbereiches möglich

### Ergebnisse der Wissensüberprüfungen

In Tab. 2 befinden sich die MW der erreichten Punktzahlen der Wissensüberprüfung. Eine Übersicht der internen Konsistenzen und der Gütekriterien ist Tab. 3 zu entnehmen. Ein Vergleich der Prüfungskohorten ist als Flussdiagramm in den Abb. 1 und 2 dargestellt.

**Table 3:** 

	TPK	ZPDT
*Theoretische Prüfung*		
Interne Konsistenz^c^	0,71	0,69
Schwierigkeit^d^	0,65	0,67
Trennschärfe^e^	0,20	0,24
*Praktische Prüfung*		
Interne Konsistenz^c^	0,84	0,49
Schwierigkeit^d^	0,57	0,60
Trennschärfe^e^	0,44	0,16
*Identische 14 Fragen*^a^		
Interne Konsistenz^c^	0,51	0,57
Schwierigkeit^d^	0,70	0,71
Trennschärfe^e^	0,19	0,24
*Identische Präparationsprüfung*^b^		
Interne Konsistenz^c^	0,77	0,66
Schwierigkeit^d^	0,60	0,60
Trennschärfe^e^	0,45	0,34

^a^Teil der theoretischen Prüfung

^b^Teil der praktischen Prüfung

^c^Mindestreliabilität (Cronbachs Alpha: 0,80; [47, 48])

^d^Arithmetisches Mittel der einzelnen Items, Richtwert 0,40–0,80 [47, 48]

^e^Arithmetisches Mittel der einzelnen Items, Richtwert 0,2–0,3 [47, 48]

**Figure 2:**
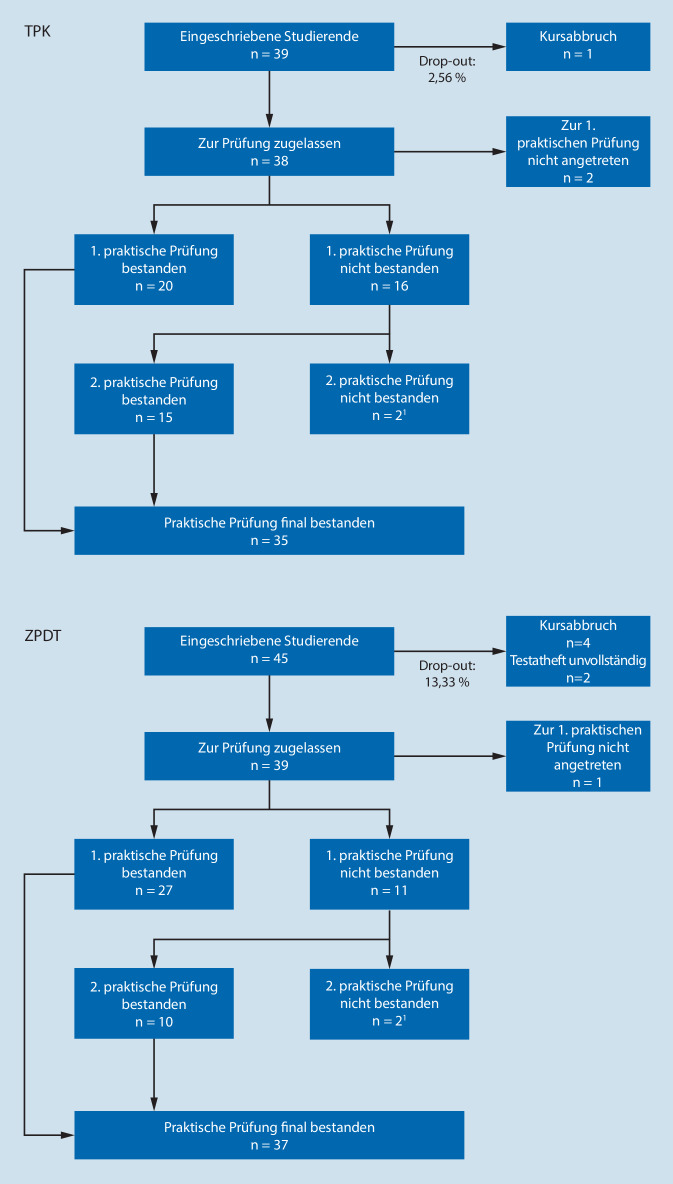

Die Durchfallquote der ersten theoretischen Prüfung betrug 29 % im TPK versus 8 % im ZPDT. Nach der Wiederholungsprüfung lagen die finalen Durchfallquoten in beiden Lehrveranstaltungen bei 0 %.

Die Durchfallquote der ersten praktischen Prüfung betrug 44 % im TPK versus 29 % im ZPDT. Die finalen Durchfallquoten lagen im TPK bei 3 % und im ZPDT bei 5 %. Am häufigsten führten die Leistungen bei der Zahnpräparation zum Nichtbestehen (11 von 16 im TPK vs. 10 von 11 im ZPDT).

Für die Gegenüberstellung der Selbsteinschätzung der Lernenden (subjektiv eingeschätzte theoretische und praktische Kompetenzen) und der Fremdeinschätzung der Lehrenden (objektiv bewertete Prüfungsergebnisse) wurden die Angaben bzgl. der Lernziele auf dem Evaluationsbogen den korrespondierenden Prüfungsergebnissen gegenübergestellt.

In ihren theoretischen Kompetenzen schätzten sich die Teilnehmenden der Lehrveranstaltung TPK zu 64,3 % übereinstimmend mit den objektiv bewerteten Prüfungsergebnissen ein, für die Teilnehmenden der Lehrveranstaltung ZPDT lag dieser Wert bei 69,6 %. Bezüglich der praktischen Kompetenzen ergab die Selbsteinschätzung der Teilnehmenden der Lehrveranstaltung TPK einen Wert von 69,8 %, während für die Lehrveranstaltung ZPDT ein Wert von 82,5 % ermittelt werden konnte.

### Generelle Rahmenbedingungen aus Sicht der Lernenden

Die fachbereichsinterne Evaluation wurde im TPK von 56 % und im ZPDT von 27 % der Lernenden vollständig ausgefüllt. Signifikante Unterschiede ergaben sich hinsichtlich der Beantwortung einzelner Fragen (siehe Tab. 2 im Onlinematerial). So wurde die Frage, ob der/die Lehrende hilfreiches Feedback auf die Beiträge der Studierenden gebe, von Lernenden des ZPDT signifikant niedriger bewertet als vom TPK (MW: 3,2 im TPK vs. 2,4 im ZPDT, *p* = 0,0285; 1 = stimme voll und ganz zu bis 6 = stimme überhaupt nicht zu). Auf der anderen Seite wurde die Frage, ob die Stoffmenge zum zeitlichen Rahmen der Veranstaltung passe, vom TPK signifikant niedriger bewertet (MW: 3,2 im TPK vs. 4,7 im ZPDT, *p* = 0,0015).

### Spezielle Lehr- und Lernbedingungen aus Sicht der Lernenden und Lehrenden

29 Lernende des TPK und 22 Lernende des ZPDT bearbeiteten den Fragebogen vollständig (Rücklaufquoten von 76 % bzw. 56 %). Die statistische Auswertung der internen Konsistenz (Cronbachs Alpha) ergab einen Wert von 0,95.

Insgesamt wurde die jeweilige Lehrveranstaltung vom TPK signifikant niedriger bewertet als vom ZPDT (MW: 2,3 im TPK vs. 2,6 im ZPDT, *p* = 0,0119; 1 = sehr gut/stimme voll zu bis 5 = mangelhaft/stimme gar nicht zu). Ebenfalls statistisch signifikante Unterschiede zeigte die getrennte Bewertung der theoretischen (MW: 2,2 im TPK vs. 2,5 im ZPDT, *p* = 0,0015) und praktischen (MW: 1,9 im TPK vs. 3,1 im ZPDT, *p* < 0,00001) Wissensvermittlung.

Aus der Subgruppenanalyse ergaben sich keine signifikanten Unterschiede hinsichtlich der Bewertung der theoretischen (Frage 12 „Situationsabformung und Modellherstellung – konventionell und digital“ mit einem MW von 1,8 im TPK vs. 2,0 im ZPDT, *p* = 0,4845) und praktischen (Frage 52 „Digitale Abformung und Design der Restauration“ mit einem MW von 2,5 im TPK vs. 3,1 im ZPDT, *p* = 0,1265) Vermittlung digitaler Kompetenzen.

Das Erreichen der lernzielbasierten Kompetenzen insgesamt wurde vom TPK und ZPDT ähnlich bewertet (MW: 2,6 im TPK vs. 2,5 im ZPDT, *p* = 0,4654). Signifikant unterschiedlich hingegen wurde die Relevanz des Anatomiekurses als Vorbereitung für die Lehrveranstaltung bewertet (MW: 2,3 im TPK vs. 1,5 im ZPDT, *p* = 0,0027). Weitere Ergebnisse befinden sich in Tab. 3 im Onlinematerial.

Der Fragebogen an die Lehrenden wurde im TPK mit einer Rücklaufquote von 90 % (9 von 10) und im ZPDT von 43 % (9 von 21) bearbeitet. Die statistische Auswertung der internen Konsistenz (Cronbachs Alpha) ergab einen Wert von 0,79. Insgesamt gab es keine signifikanten Unterschiede in der Evaluation durch die Lehrenden. Jedoch wurden Fragen, in denen es um die zur Verfügung stehende Zeit im Rahmen der Lehrveranstaltung ging, für den TPK signifikant niedriger bewertet als für den ZPDT (Frage 32 „Die curricular vorgegebene Anzahl von im Mittel … SWS für das Praktikum war zum Erlernen der praktischen Fertigkeiten angemessen“ mit einem MW von 1,4 im TPK vs. 4,2 im ZPDT, *p* = 0,0004). Weitere Ergebnisse zu den einzelnen Fragen befinden sich in Tab. 4 im Onlinematerial.

Bezüglich der Vergleichsparameter 1, 2 und 4 gab es in der Gesamtbetrachtung keine signifikanten Unterschiede zwischen beiden Veranstaltungen. Der Vergleichsparameter 3 wurde hingegen insgesamt signifikant niedriger von Lernenden des TPK-Kurses bewertet.

## Diskussion

Die Evaluation von Lehrveranstaltungen ist in der Literatur umfangreich beschrieben [5–11, 32]. In der vorliegenden Studie wurden quantitative (Prüfungen und Fragebögen) und qualitative (Freitextkommentare) Daten erhoben. Zusätzlich erfolgte die Evaluation unterschiedlicher Parameter. Die Erhebung und Interpretation dieser Daten ermöglichten ein wie von Fraenkel et al. beschriebenes [5] trianguliertes Design der Evaluation. Im Rahmen dieser Studie wurden die Evaluationsinstrumente zur Erfassung der speziellen Lehr- und Lernbedingungen (Parameter 3 und 4) neu entwickelt und sowohl Lernende als auch Lehrende einbezogen.

Bezüglich der Wissensprüfungen finden sich ähnliche finale Durchfallquoten in der Literatur (5 % für theoretische und 1 % für praktische Prüfungen; [33]). Bezüglich der gemeinsamen Prüfungsteile (14 identische Fragen in der Theorie, Präparationsprüfung in der Praxis) bestanden zwar keine signifikanten Unterschiede, jedoch fällt auf, dass Lernende des ZPDT final häufiger wegen der Präparationsprüfung durchfielen als Lernende des TPK. Dies könnte damit zusammenhängen, dass die Studierenden im TPK länger die Möglichkeiten hatten, Präparationsübungen vor Ort durchzuführen.

Die Prüfungen waren nahezu identisch. Lediglich die Theorieprüfung wurde im TPK analog und im ZPDT digital auf Tablets bearbeitet. Anlehnend an Studien von Bloomfield kann dieser Unterschied ebenfalls zu Problemen in der Vergleichbarkeit führen [34]. Alle anderen Evaluationsinstrumente (Parameter 2–4) wurden hingegen identisch implementiert.

Die Gegenüberstellung der Selbst- und Fremdeinschätzung beider Kohorten bzgl. der theoretischen und praktischen Kompetenzen unterschied sich nur geringfügig und war nicht signifikant. Vergleichbare Gegenüberstellungen wurden bereits für Studierende der Humanmedizin beschrieben [35].

Die Rücklaufquoten der Parameter 2 bis 4 (74 % im TPK vs. 42 % im ZPDT) lagen vor dem Hintergrund des verwendeten Online-Befragungsmodus nah am durchschnittlichen Rücklauf von 44,1 %, der in der Publikation von Wu et al. beschrieben wurde [36]. Um die Rücklaufquote der Evaluation der speziellen Lehr- und Lernbedingungen aus Sicht der Lernenden (Parameter 3) zu erhöhen, wurde jeweils in beiden Veranstaltungen eine zweite und dritte analoge Runde durchgeführt, orientierend an den Veröffentlichungen von Cottrell et al. sowie Weston et al. [37, 38].

Es gab zahlreiche signifikante Unterschiede in der Evaluation der Lehrveranstaltung TPK und ZPDT (siehe Tab. 3 im Onlinematerial). Auch hier gibt es möglicherweise einen Zusammenhang zu dem unterschiedlichen zeitlichen Rahmen der Lehrveranstaltungen vor Ort: TPK-Studierende hatten ca. 4‑mal so viel Zeit für die Lehrveranstaltung verglichen mit Studierenden des ZPDT (280 SWS vs. 70 SWS). Dafür wurde erstmalig im ZPDT ein Selbststudium, inkl. Feedback, eingeführt. Die Gesamtkurszeit betrug somit 280 Semesterstunden im TPK vs. 197 im ZPDT (Tab. 1). Nichtsdestotrotz wurde der Faktor „Zeit“ auch in den Freitextantworten aufgenommen (Tab. 4). Als wesentliche Punkte in diesem Kontext wurden Probleme in Bezug auf „Praktika“ und „Betreuung“ erwähnt, deren Relevanz auch in der Publikation von Nasser et al. beschrieben wurde [39]. Diese Erkenntnisse unterstreichen die Wichtigkeit von regelmäßigem Feedback in Zusammenhang mit dem Selbststudium. Die Auswirkung des Feedbackzeitpunktes wurde von Kehrer et al. untersucht [40]. Die hohe Bedeutung des Feedbacks für Zahnmedizinstudierende in curricularen Lehrveranstaltungen wurde bereits von Zaric et al. beschrieben [41].

**Table 4:** 

		TPK	ZPDT
Lernende	Eher positiv	„Folien der Vorlesungen wurden immer hochgeladen“	„Gute Verbindung zwischen Praxis und Theorie“
		„Freundlicher Umgang“	„Gute Betreuung durch ÄrztInnen“
		„Gute Lernatmosphäre der Studierenden“	„Individuelle Fragen wurden auch berücksichtigt und beantwortet“
		„Die ÄrztInnen im … waren hilfsbereit …“	„Teilweise sehr engagierte Lehrende“
		„Die Vorlesungen wurden strukturiert gestaltet und die Folien wurden rechtzeitig zur Verfügung gestellt“	„Theoretischen Bezug zu praktischen Übungen“
		„Vorlesungen oft interaktiv gestaltet“	„Vorlesungen waren meistens etwas früher hochgeladen, sodass die Folien davor schon grob angeschaut werden konnten, um sich vorzubereiten“
		„… sehr hilfreich, dass im … während dem Präparieren gleichzeitig die Videos zum Präparieren auf den Bildschirmen liefen“	„Klare Verbesserungsvorschläge bei praktischen Tätigkeiten …“
		„… immer eine kurze Erklärung von den ZahntechnikerInnen bekommen worauf genau geachtet werden soll“	„… auf Fragen wurde immer eingegangen …“
		„Schneller Wissenszuwachs und schnelles Erlernen der praktischen Arbeiten“	„… TutorInnen waren sehr hilfreich …“
		„Die TutorInnen während der Zeit des freien Übens waren sehr hilfreich“	„Technische Fertigkeiten“
		„Der inhaltliche Umfang der Vorlesungen ist gut machbar“	„Erlernen des Umgangs mit Stresssituationen“
		„Erlernen zahntechnischer und zahnmedizinischer Vorgänge“	„Sehr abwechslungsreich …“
	Eher negativ	„Diskrepanz & Widerspruch der ÄrztInnen hinsichtlich der Bewertungskriterien“	„… Überziehen der Vorlesungen so gering wie möglich halten“
		„Mehr Seminararbeit, Diskussionen eventuell?“	„… klausurrelevante Themen verdeutlichen, sodass man besser nacharbeiten kann“
		„Intensiveres Feedback der Gelehrten …“	„Mehr Zeit für die praktischen Übungen (vor allem fürs Präparieren)“
		„Mehr Personen, die testieren dürfen …“	„Mehr Zeit für Testate“
		„Mehr Equipment …“	„Mehr Übungszeit im Kurs“
		„Die Vorlesungen waren eine sehr gute Grundlage für das praktische Arbeiten, trotzdem hätte mir eine Demo von der praktischen Arbeit im Simulationslabor wahrscheinlich sehr weiter geholfen …“	„Praktische Beispiele, z. B. auch Vorzeigen im Kurs. Positiv- sowie Negativbeispiele zur Orientierung“
		„Intensiveres Feedback …“	„Wesentlich mehr Zeit zum Ausführen der praktischen Arbeiten“
		„Bessere Kommunikation“	„Eindeutigere Kommunikation …“
		„Einheitliche Anforderungen an alle“	„Viel zu wenig Zeit …“
		„… weniger Stunden, die dafür effektiver genutzt werden …“	„Mehr Praktikumstage und wenn möglich für die Präparationshausaufgabe das Lern-Lab zur Verfügung stellen …“
		„… mehr Demos von ÄrztInnen während des Kurses …“	„Demos immer mit praktischer Vorführung“
		„Vorlesungen interessanter gestalten …“	„Mehr Betreuung durch AssistenzärztInnen beim Präparieren“
Lehrende	Eher positiv	k. A.	k. A.
	Eher negativ	„Mehr digitale Abformung und Design der Restauration“	„Mehr Semesterwochenstunden im Praktikum wären wünschenswert“
			„Mehr Motivation erwünscht zu Besuch der Vorlesungen“
			„Rüstzeit verlängern für Wechsel zwischen Gruppen“
			„Lernziele ZPDT & ZPPZ auf Doppelungen überprüfen“
			„Regelmäßige Wissensüberprüfung im Rahmen der begleitenden Seminare einführen“
			„Reinigungsdienstplan einführen“

*k. A.* keine Angaben, *ZPPZ* Zahnmedizinische Propädeutik mit Schwerpunkt Präventive Zahnheilkunde

Die Selbsteinschätzung der Kompetenzen im Abschnitt „Lernziele“ unterschied sich in beiden Kohorten insgesamt nicht. Signifikant niedriger (wobei 1 = sehr gut/stimme voll zu bis 5 = mangelhaft/stimme gar nicht zu) waren jedoch die Selbsteinschätzungen der Lernenden des ZPDT in Bezug auf kieferorthopädische und implantologische Kompetenzen (Lernziele 97 und 101). Diese Ergebnisse könnten Ausdruck der systematischen Umsetzung der NKLZ-basierten Lernziele und der daraus resultierenden Interdisziplinarität im ZPDT sein. Dabei spielt die neue Approbationsordnung (ZApprO) eine entscheidende Rolle für die entsprechende Implementierung, wie auch in der Veröffentlichung von Kahl-Nieke et al. beschrieben [3].

Fragen, die sich auf Aspekte der Digitalisierung beziehen (digitale Abformung, CAD[„Computer-Aided Design“]/​CAM[„Computer-Aided Manufacturing“]-Prinzipien), wurden in beiden Lehrveranstaltungen ähnlich bewertet. Dies lässt sich am ehesten damit erklären, dass diese schon in der Lehrveranstaltung TPK implementiert wurden, noch bevor die ZApprO in Kraft trat.

Der Nutzen eines Anatomiekurses vor der Lehrveranstaltung wurde im ZPDT signifikant niedriger bewertet als im TPK. Dies könnte mit dem zeitlichen Kontext begründet werden. Im TPK fand dieser Kurs verteilt auf 3 Semester statt, im ZPDT unmittelbar vor der Lehrveranstaltung in Form eines Einführungskurses. Der Nutzen von Anatomiekursen für Zahnmedizinstudierende generell wird auch in der Literatur beschrieben [42, 43].

Die fachbereichsinterne Evaluation und die der Lehrenden unterschieden sich insgesamt in beiden Kursen nicht. Einzelne Ergebnisse spiegeln jedoch die Einschätzung der Lernenden des ZPDT wider, wonach die für die Lehrveranstaltung zur Verfügung stehende Zeit als zu niedrig empfunden wurde.

### Limitationen

Die Teilnehmenden der vorliegenden Studie gehörten 2 unterschiedlichen Semestern (Kohorten) an. Weiterhin erfolgte keine randomisierte Zuteilung. Die Lernenden des TPK waren zudem im Schnitt 4 Jahre älter als die des ZPDT, hatten bereits mindestens 4 Semester des Zahnmedizinstudiums absolviert und befanden sich somit nicht mehr in Regelstudienzeit. Diese Limitation war durch die unterschiedlichen Studienordnungen vorgegeben. Eine entsprechende Harmonisierung wurde in mehreren Studien gefordert, dies war jedoch in der vorliegenden Untersuchung nicht umsetzbar [5, 44, 45]. Die genannten Faktoren gehen mit unterschiedlichen Erfahrungen mit dem Studium der Zahnmedizin einher. Die Studierenden des TPK verfügen über mehr Lebens- und Bildungserfahrung, was sich auf ihre Fähigkeiten, ihr Wissen oder ihre Motivation auswirken könnte. Dies limitiert die Vergleichsmöglichkeit und Generalisierbarkeit.

Ein Abbruch der Lehrveranstaltung erfolgte durch 1 (3 %) TPK- bzw. 4 (9 %) ZPDT-Lernende. In keinem der Fälle wurde der Abbruch begründet, die Lernenden nahmen weder an der Prüfung noch an den Evaluationen teil. Lernende, die nicht zur Prüfung zugelassen wurden oder nicht antraten, verringerten die Fallzahl ebenfalls. Dies traf jedoch nur auf den ZPDT zu (2 von 45, 4 %).

Die in der Studie verglichenen Lehrveranstaltungen fanden zu unterschiedlichen Zeiten statt (ZPDT: April bis Juli 2022, TPK: August bis September 2022). Dementsprechend fanden die Prüfungen und die fachbereichsinterne Evaluation ebenfalls nicht zeitgleich statt. Die hier evaluierte Lehrveranstaltung TPK wurde letztmalig durchgeführt, sodass erneute Vergleichsmöglichkeiten entfallen. Da die Lehrinhalte jedoch auch von den Studienordnungen der Universitäten abhängen [4], können die Lehrveranstaltungen TPK und ZPDT zwischen den Standorten variieren. Um die Generalisierbarkeit der Evaluationsergebnisse der Lehrveranstaltung ZPDT zu erhöhen, sind zukünftige multizentrische Untersuchungen empfehlenswert [46].

### Fazit

Am Universitätsstandort Frankfurt am Main erfolgte ein Vergleich der Lehrveranstaltung „Zahnmedizinische Propädeutik mit Schwerpunkt Dentale Technologie“ (ZPDT), die nach der aktuellen Approbationsordnung (ZApprO) im ersten Studienabschnitt implementiert wurde, mit der Lehrveranstaltung „Kurs der Technischen Propädeutik“ (TPK), die entsprechend der alten Approbationsordnung für Zahnärzte (ZÄPrO) vor der zahnärztlichen Vorprüfung stattfand. Der Kohortenvergleich zeigte eine ausgewogene Bewertung der Parameter „Wissensüberprüfung“, „fachbereichsinterne Evaluation zur Erfassung genereller Rahmenbedingungen aus Sicht der Lernenden“ sowie „Evaluation zur Erfassung spezieller Lehr- und Lernbedingungen aus Sicht der Lehrenden“. Lediglich die „Evaluation zur Erfassung spezieller Lehr- und Lernbedingungen aus Sicht der Lernenden“ fiel signifikant zugunsten des TPK aus, insbesondere im Hinblick auf die theoretische und praktische Wissensvermittlung. Die hier formulierte Hauptforschungsfrage, ob es bezüglich der untersuchten Parameter Unterschiede zwischen den beiden Lehrveranstaltungen gibt, muss somit differenziert beantwortet werden: Es zeigt sich eine Diskrepanz zwischen subjektiver Beurteilung der Wissensvermittlung und objektiv bewerteten Prüfungsergebnissen. Letztere könnten Ausdruck der neu implementierten Selbststudienzeiten in Kombination mit korrektivem Feedback im ZPDT sein. Diese sollten aus Sicht der Autorenschaft weiterhin angeboten werden. Für die Zukunft erscheint es relevant, die zahnmedizinische Ausbildungsforschung auf konkrete Faktoren zu fokussieren, die die Ausbildungsqualität der Lernenden möglicherweise beeinflussen, wie z. B. die benannten Selbststudienzeiten oder die Bedeutung von gezieltem Feedback. Der fakultätsübergreifende Einsatz der speziell für die Anwendung im Rahmen der ZApprO entwickelten Evaluationsinstrumente könnte dabei hilfreich sein.

### Supplementary Information




