# Supplementary material for: ZApprO versus ZÄPrO: results of a first comparative study
Source: Bundesgesundheitsblatt Gesundheitsforschung Gesundheitsschutz. 2023 Oct 20;66(12):1397–408. [Article in German] doi: 10.1007/s00103-023-03788-z (PMC10667395; doi:10.1007/s00103-023-03788-z)
Supplement: Supplementary file 1 [file 103_2023_3788_MOESM1_ESM.docx]

**Onlinematerial**

**Tabelle 1 – Ein- und Ausschlusskriterien**

|  | **TPK** | **ZPDT** |
| --- | --- | --- |
| **Einschlusskriterien** | Studierende des 5. und 6. Fachsemesters | Studierende des 1. Fachsemesters |
|  | Studierende, die nach der alten Approbationsordnung studieren | Studierende, die nach der neuen Approbationsordnung studieren |
|  | Studierende, die die Lehrveranstaltung erstmalig besuchen oder wiederholen | Studierende, die die Lehrveranstaltung erstmalig besuchen |
| **Ausschlusskriterien** | Studierende aller anderen Fachsemester | Studierende aller anderen Fachsemester |
|  | Studierende, die nach der neuen Approbationsordnung studieren | Studierende, die nach der alten Approbationsordnung studieren |
|  |  | Studierende, die die Lehrveranstaltung wiederholen |

TPK = Kurs der Technischen Propädeutik; ZPDT = Zahnmedizinische Propädeutik mit Schwerpunkt Dentale Technologie


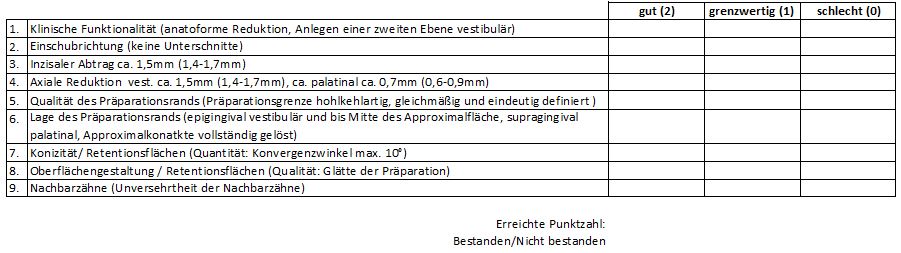
**Abbildung 1 – Bewertungskriterien praktische Prüfung, Präparationsteil (TPK und ZPDT)**

(Quelle: Eigene Abbildung)


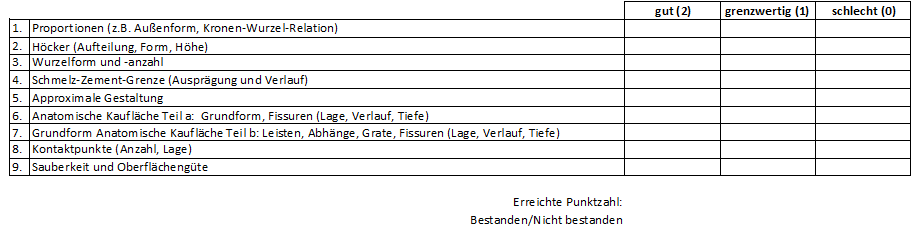
**Abbildung 2 – Bewertungskriterien praktische Prüfung, Modellationsteil (Knete) im TPK**

(Quelle: Eigene Abbildung)


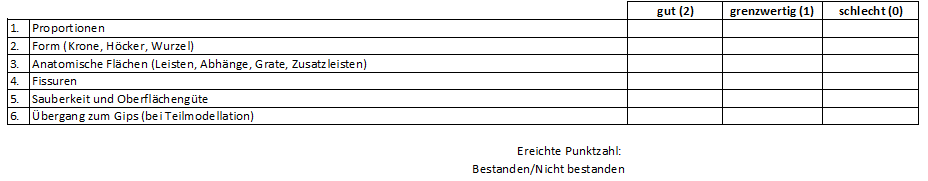
**Abbildung 3 – Bewertungskriterien praktische Prüfung, Modellationsteil (Wachs) im ZPDT**

(Quelle: Eigene Abbildung)


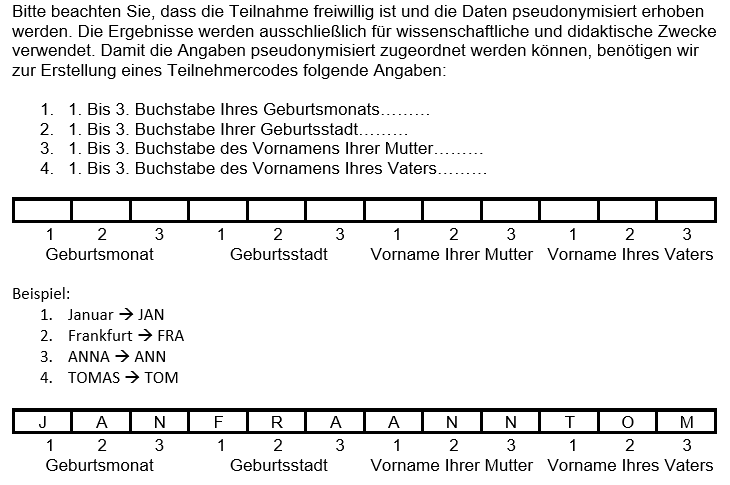
**Abbildung 4 – Angaben zur Pseudonymisierung im Rahmen der Evaluation zur Erfassung spezieller Lehr- und Lernbedingungen durch die Lernenden, 3. Parameter**

(Quelle: Eigene Abbildung)


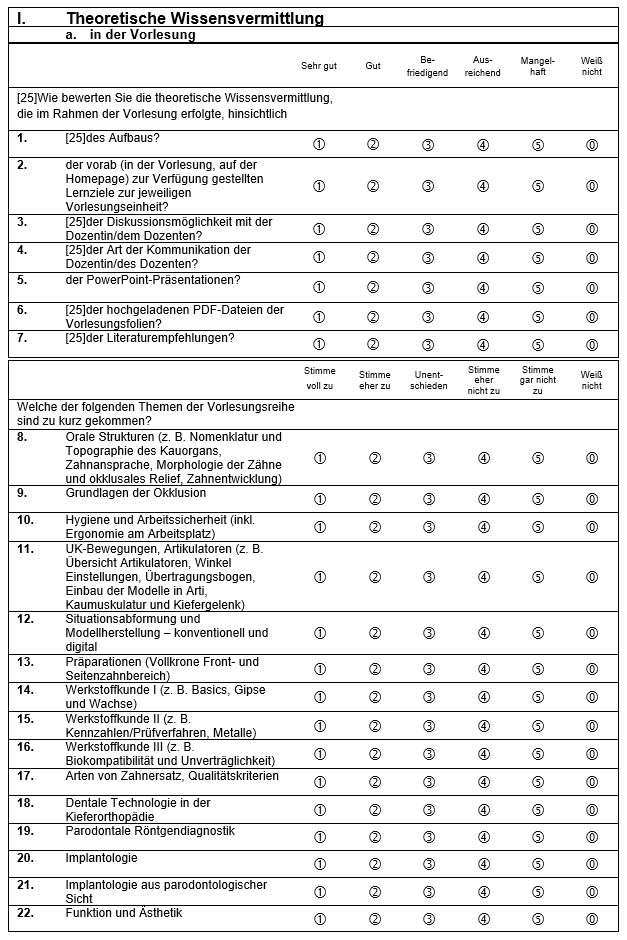
**Abbildung 5 – Fragebogen zur Erfassung spezieller Lehr- und Lernbedingungen aus Sicht der Lernenden (3. Parameter) mit Verweisen ins Literaturverzeichnis ^a^, Seite 1 von 6**

^a^ Erklärung zu Abb. 5-12: Die Angaben in den eckigen Klammern verweisen auf korrespondierende Publikationen aus dem Literaturverzeichnis. (Quelle: Eigene Abbildung).


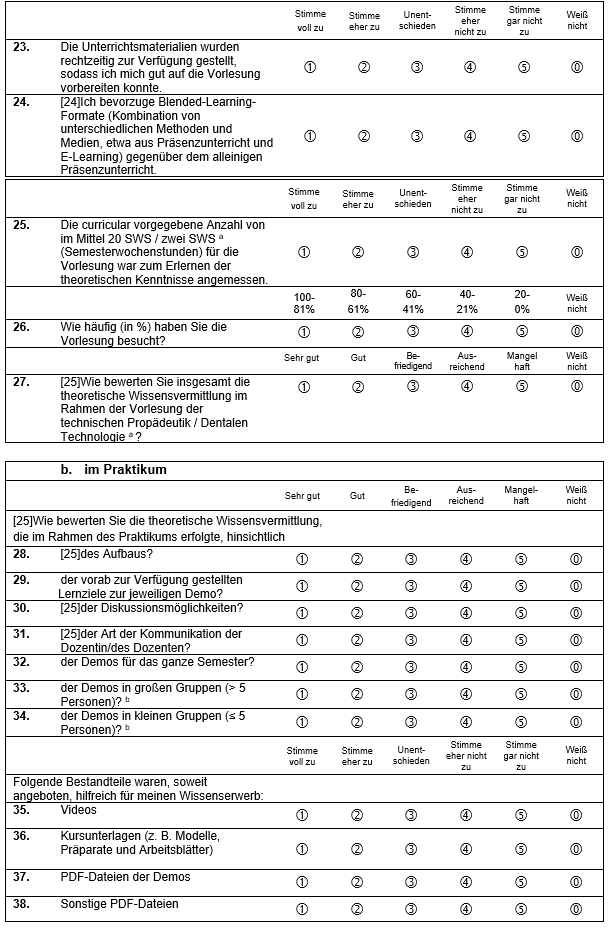
**Abbildung 6 – Fragebogen zur Erfassung spezieller Lehr- und Lernbedingungen aus Sicht der Lernenden (3. Parameter) mit Verweisen ins Literaturverzeichnis, Seite 2 von 6**

^a^ An dieser Stelle differierte die Frage im Fragebogen an den TPK (links) bzw. an den ZPDT (rechts): (TPK/ZPDT).

^b^ Fragen 33 und 34 wurden lediglich für die Lehrveranstaltung ZPDT abgefragt.


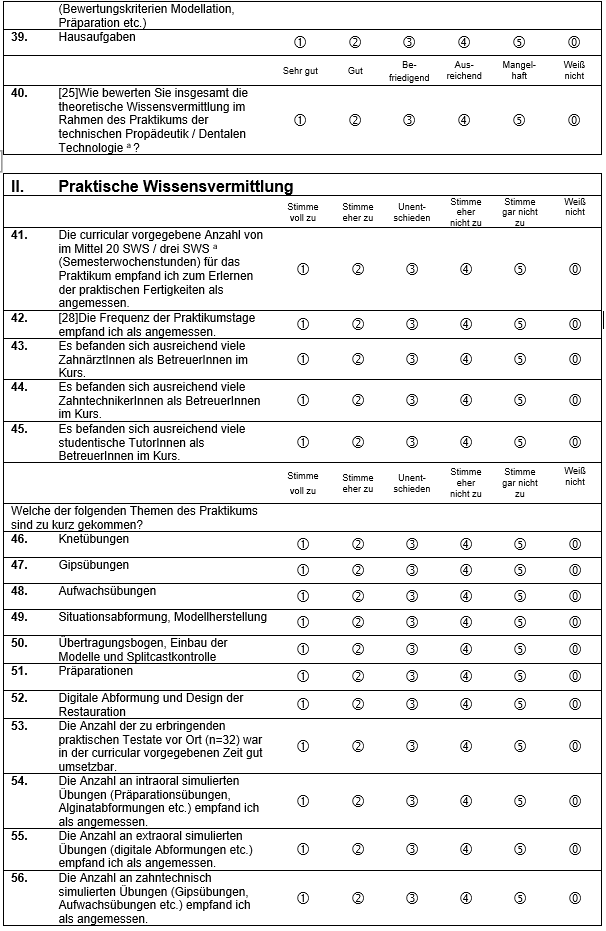
**Abbildung 7 – Fragebogen zur Erfassung spezieller Lehr- und Lernbedingungen aus Sicht der Lernenden (3. Parameter) mit Verweisen ins Literaturverzeichnis, Seite 3 von 6**

^a^ An dieser Stelle differierte die Frage im Fragebogen an den TPK (links) bzw. an den ZPDT (rechts): (TPK/ZPDT).


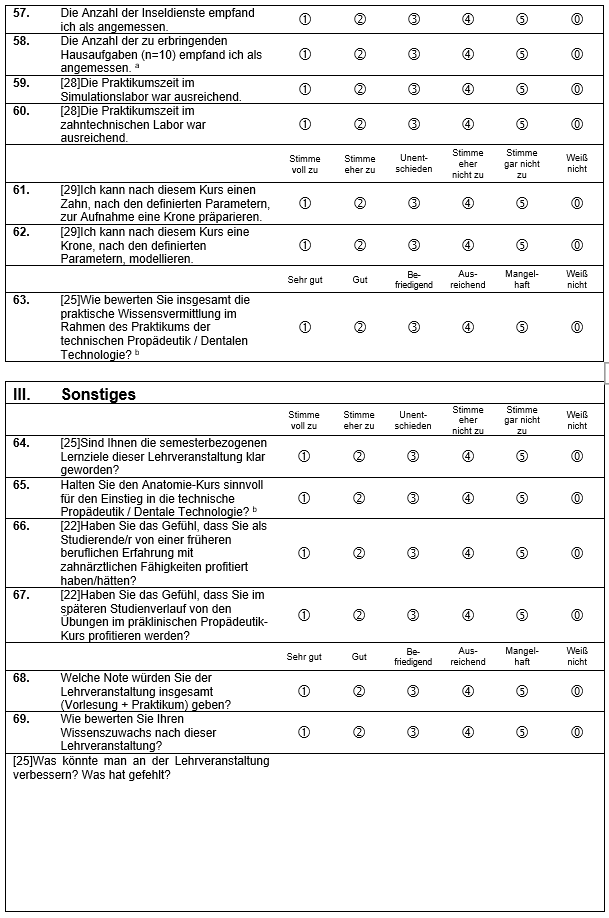
**Abbildung 8 – Fragebogen zur Erfassung spezieller Lehr- und Lernbedingungen aus Sicht der Lernenden (3. Parameter) mit Verweisen ins Literaturverzeichnis, Seite 4 von 6**

^a^ Frage 58 wurde lediglich für die Lehrveranstaltung ZPDT abgefragt.

^b^ An dieser Stelle differierte die Frage im Fragebogen an den TPK (links) bzw. an den ZPDT (rechts): (TPK/ZPDT).


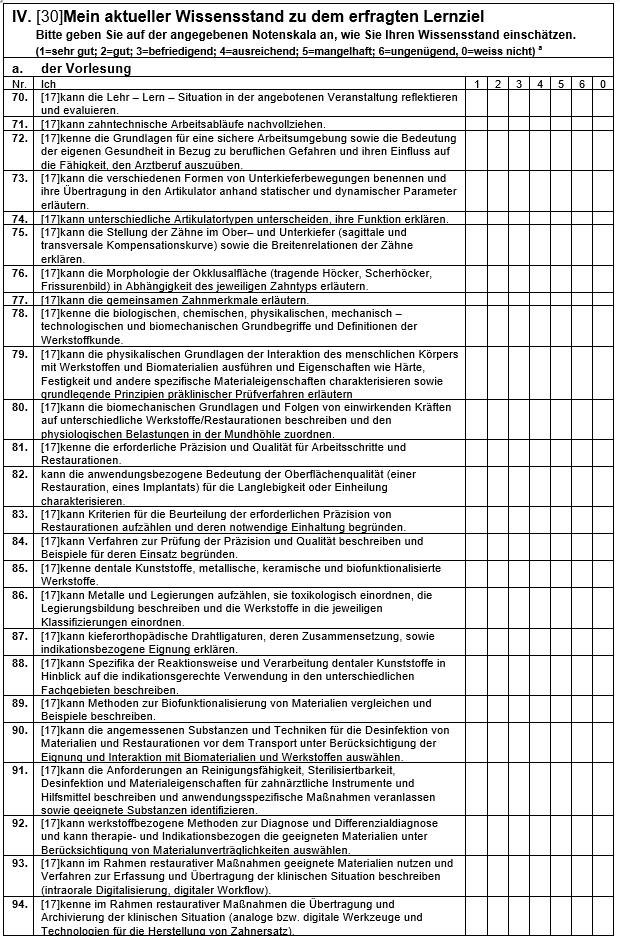
**Abbildung 9 – Fragebogen zur Erfassung spezieller Lehr- und Lernbedingungen aus Sicht der Lernenden (3. Parameter) mit Verweisen ins Literaturverzeichnis, Seite 5 von 6**

^a^ Die Skala wurde von 1-6 auf 1-5 umkodiert, um eine Angleichung der Skalen zum restlichen Fragebogen zu erreichen.


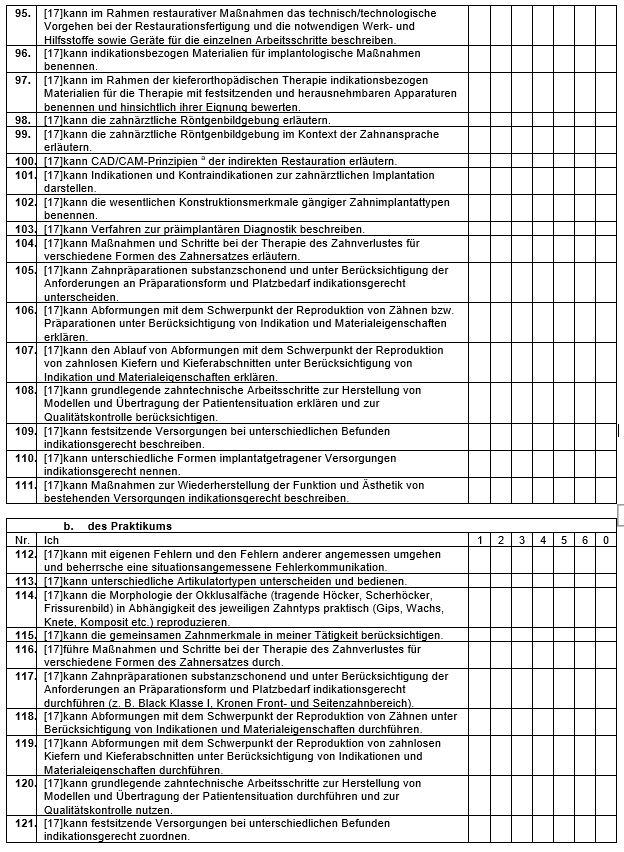
**Abbildung 10 – Fragebogen zur Erfassung spezieller Lehr- und Lernbedingungen aus Sicht der Lernenden (3. Parameter) mit Verweisen ins Literaturverzeichnis, Seite 6 von 6**

^a^ CAD/CAM = Computer-Aided Design/Computer-Aided Manufacturing


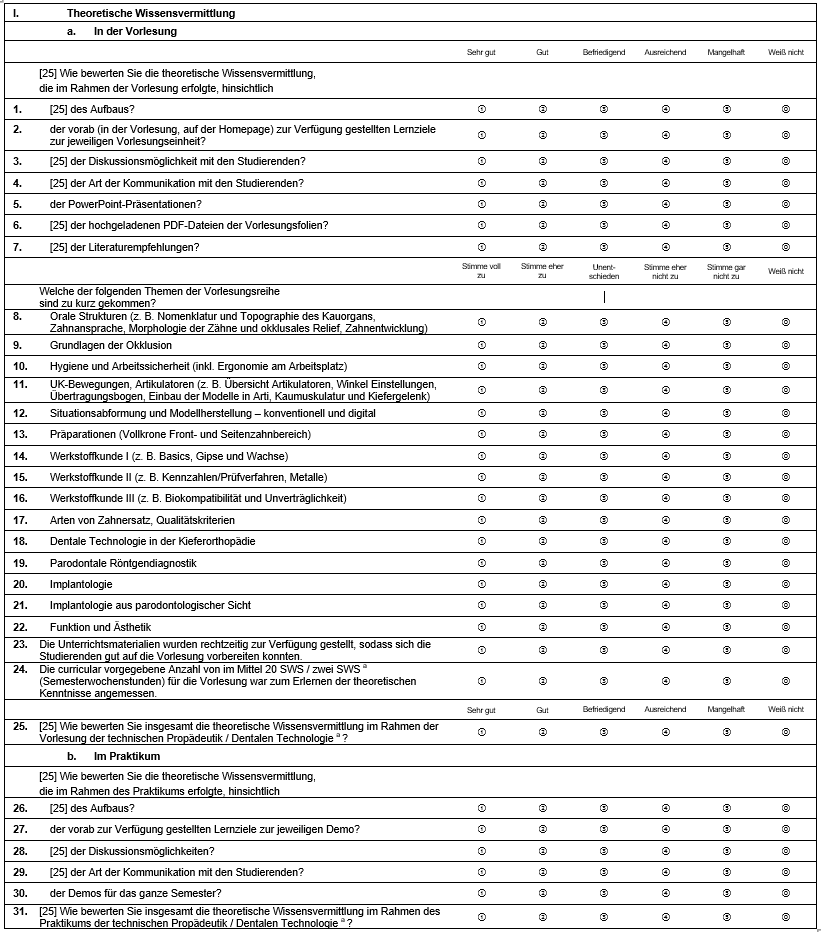
**Abbildung 11 – Fragebogen zur Erfassung spezieller Lehr- und Lernbedingungen aus Sicht der Lehrenden (4. Parameter) mit Verweisen ins Literaturverzeichnis, Seite 1 von 2**

^a^ An dieser Stelle differierte die Frage im Fragebogen an den TPK (links) bzw. an den ZPDT (rechts): (TPK/ZPDT).

**Abbildung 12 – Fragebogen zur Erfassung spezieller Lehr- und Lernbedingungen aus Sicht der**
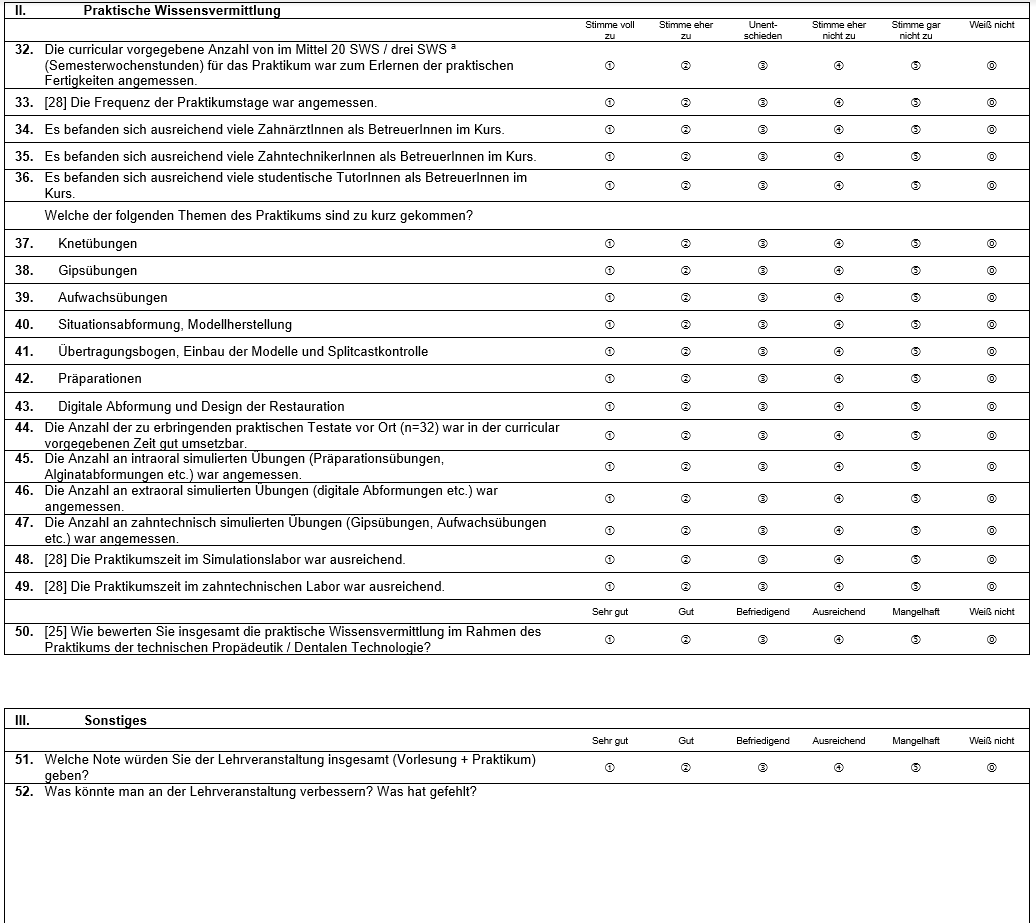
**Lehrenden (4. Parameter) mit Verweisen ins Literaturverzeichnis, Seite 2 von 2**

^a^ An dieser Stelle differierte die Frage im Fragebogen an den TPK (links) bzw. an den ZPDT (rechts): (TPK/ZPDT)

**Tabelle 2 – Ergebnisse der fachbereichsinternen Evaluation zur Erfassung genereller Rahmenbedingungen aus Sicht der Lernenden (Parameter Nr. 2 ^a^)**

|  | **TPK** | **ZPDT** | **p-Wert** |
| --- | --- | --- | --- |
| **I. Angaben zur Organisation** |  |  |  |
| 1. Die in der Veranstaltung genutzten Lehrmaterialien sind angemessen. | 2,70±1,30 | 3,00±1,00 | 0,3871 |
| 1. Die Lehrmaterialien werden zur Verfügung gestellt (z. B. in OLAT). | 2,20±1,00 | 1,70±1,10 | 0,1685 |
| 1. Die Lehrveranstaltung fand statt. | 2,20±0,80 | 1,30±0,70 | 0,0010* |
| 1. Das angekündigte Thema wurde gelehrt. | 2,20±1,00 | 1,70±1,00 | 0,1369 |
| 1. Der/die Lehrende war pünktlich. | 2,50±1,10 | 2,60±1,10 | 0,7797 |
| **II. Angaben zur Lehrveranstaltung** |  |  |  |
| 1. Der Besuch der Veranstaltung führt zu einem spürbaren Wissenszuwachs. | 2,70±1,20 | 2,80±1,00 | 0,7668 |
| 1. Inhalte werden anschaulich vermittelt. | 3,20±1,30 | 2,90±1,00 | 0,3871 |
| 1. In der Veranstaltung werden auch schwierige Inhalte verständlich erklärt. | 3,40±1,30 | 3,30±1,20 | 0,8005 |
| 1. Die Relevanz der behandelten Themen wird deutlich. | 2,80±1,00 | 2,60±0,80 | 0,4655 |
| 1. Der/die Lehrende ist in der Lage, strukturiert zu erklären. | 2,90±1,30 | 2,30±0,90 | 0,0797 |
| 1. Der/die Lehrende gibt hilfreiches Feedback auf die Beiträge der Studierenden. | 3,20±1,30 | 2,40±1,00 | 0,0285* |
| 1. Der/die Lehrende achtet darauf, eine wertschätzende Lehr-/Lernatmosphäre herzustellen. | 3,60±1,40 | 3,40±2,00 | 0,7576 |
| 1. Meine Mitstudierenden tragen zu einer konstruktiven Lernatmosphäre bei. | 2,40±1,20 | 1,50±0,50 | 0,0002* |
| 1. Die Lehrveranstaltung folgt einer klaren Struktur. | 3,10±1,20 | 2,60±0,50 | 0,0275* |
| 1. Der/die Lehrende war auf die Veranstaltung vorbereitet. | 2,30±0,90 | 1,70±0,90 | 0,0530 |
| 1. Die Stoffmenge passt zum zeitlichen Rahmen der Veranstaltung. | 3,20±1,60 | 4,70±1,20 | 0,0015* |
| 1. In der Veranstaltung werden Lernziele oder Take-Home-Messages dargestellt. | 3,00±1,30 | 2,50±1,10 | 0,1862 |
| **III. Ergänzung Digitale Lehre** |  |  |  |
| 1. Ich habe ausreichend Informationen zur Nutzung des digitalen Lehr-/Lernangebots von dem/der Lehrenden erhalten. | 2,50±1,20 | 2,30±0,90 | 0,5621 |
| 1. In der Bedienung der Online-Lernumgebung habe ich mich schnell zurechtgefunden. | 1,90±0,90 | 2,10±0,80 | 0,5151 |
| 1. Das Lernen mit dem digitalen Lehr-/Lernangebot lief technisch einwandfrei. | 2,20±0,90 | 2,60±1,10 | 0,3032 |
| 1. Mit Hilfe des digitalen Lehr-/Lernangebots konnte ich die Lernziele der Lehrveranstaltung gut erreichen. | 2,40±1,10 | 2,50±1,00 | 0,7829 |
| 1. Die investierte Zeit in die Bearbeitung des digitalen Lehr-/Lernangebots ist im Verhältnis zum Lernerfolg angemessen. | 2,30±1,10 | 2,50±0,70 | 0,4812 |
| 1. Ich habe das digitale Lehr-/Lernangebot intensiv genutzt (d. h. alle bzw. fast alle Online-Themen und -Aufgaben aktiv bearbeitet) | 2,20±1,10 | 1,90±0,70 | 0,2943 |
| 1. Die innerhalb des digitalen Lehr-/Lernangebots gestellten Aufgaben (Übungen, Forendiskussion etc.) waren verständlich und klar formuliert. | 2,60±1,10 | 2,70±1,20 | 0,8238 |
| 1. Ich hatte ausreichend Möglichkeiten, dem/der Lehrenden Rückfragen zum digitalen Lehr-/Lernangebot zu stellen. | 3,30±1,50 | 2,70±1,00 | 0,1766 |
| 1. Insgesamt bin ich mit dem digitalen Lehr-/Lernangebot zufrieden. | 2,70±1,40 | 2,40±1,00 | 0,4418 |

^a^ 1 = stimme voll und ganz zu – 6 = stimme überhaupt nicht zu

TPK = Kurs der Technischen Propädeutik; ZPDT = Zahnmedizinische Propädeutik mit Schwerpunkt Dentale Technologie; OLAT = Lernplattform Online Learning and Training.

* Statistische Signifikanz (p< ≤0,05)

**Tabelle 3 – Ergebnisse der Evaluation zur Erfassung spezieller Lehr- und Lernbedingungen aus Sicht der Lernenden (Parameters Nr. 3 ^a^)**

|  | **TPK ^b^** | **ZPDT ^c^** | **p-Wert** |
| --- | --- | --- | --- |
| **II. Theoretische Wissensvermittlung** |  |  |  |
| 1. **In der Vorlesung** |  |  |  |
| Wie bewerten Sie die theoretische Wissensvermittlung, die im Rahmen der Vorlesung erfolgte, hinsichtlich |  |  |  |
| 1. des Aufbaus? | 1,97±0,57 | 2,64±1,05 | 0,0108* |
| 1. der vorab (in der Vorlesung, auf der Homepage) zur Verfügung gestellten Lernziele zur jeweiligen Vorlesungseinheit? | 2,46±1,07 | 2,27±0,83 | 0,4789 |
| 1. der Diskussionsmöglichkeit mit der Dozentin/dem Dozenten? | 1,76±0,69 | 2,55±0,96 | 0,0025* |
| 1. der Art der Kommunikation der Dozentin/des Dozenten? | 1,72±0,92 | 2,36±0,85 | 0,0134* |
| 1. der PowerPoint-Präsentationen? | 1,97±0,82 | 2,32±0,72 | 0,1091 |
| 1. der hochgeladenen PDF-Dateien der Vorlesungsfolien? | 2,11±0,83 | 2,00±0,71 | 0,6290 |
| 1. der Literaturempfehlungen? | 2,62±0,94 | 2,29±1,05 | 0,3138 |
| Welche der folgenden Themen der Vorlesungsreihe sind zu kurz gekommen? ^d^ |  |  |  |
| 1. Orale Strukturen (z. B. Nomenklatur, Topografie Kauorgan, Zahnansprache, Morphologie Zähne, okklusales Relief, Zahnentwicklung) | 2,14±1,22 | 1,68±0,57 | 0,0824 |
| 1. Grundlagen der Okklusion | 2,17±1,14 | 3,59±1,10 | 0,0000* |
| 1. Hygiene und Arbeitssicherheit (inkl. Ergonomie am Arbeitsplatz) | 1,83±1,14 | 1,55±0,74 | 0,2892 |
| 1. UnterkieferbBewegungen, Artikulatoren (z. B. Artikulatoren, Winkel, Übertragungsbogen, Einbau der Modelle, Kaumuskulatur, Kiefergelenk) | 2,31±1,20 | 3,05±1,16 | 0,0341* |
| 1. Situationsabformung und Modellherstellung – konventionell und digital | 1,83±1,04 | 2,05±1,13 | 0,4845 |
| 1. Präparationen (Vollkrone Front- und Seitenzahnbereich) | 1,97±1,24 | 2,36±1,29 | 0,2734 |
| 1. Werkstoffkunde I (z. B. Basics, Gipse und Wachse) | 1,89±0,96 | 2,64±1,09 | 0,0156* |
| 1. Werkstoffkunde II (z. B. Kennzahlen/Prüfverfahren, Metalle) | 1,86±0,89 | 2,76±1,14 | 0,0046* |
| 1. Werkstoffkunde III (z. B. Biokompatibilität und Unverträglichkeit) | 1,93±0,98 | 2,57±1,16 | 0,0477* |
| 1. Arten von Zahnersatz, Qualitätskriterien | 2,28±1,16 | 2,32±1,04 | 0,8918 |
| 1. Dentale Technologie in der Kieferorthopädie | 2,81±1,13 | 2,82±1,33 | 0,9769 |
| 1. Parodontale Röntgendiagnostik | 3,28±1,31 | 3,09±1,31 | 0,6231 |
| 1. Implantologie | 3,42±1,14 | 2,76±1,14 | 0,0608 |
| 1. Implantologie aus parodontologischer Sicht | 3,33±1,17 | 2,81±1,36 | 0,1773 |
| 1. Funktion und Ästhetik | 2,74±1,16 | 2,14±1,06 | 0,0702 |
| 1. Die Unterrichtsmaterialien wurden rechtzeitig zur Verfügung gestellt, sodass ich mich gut auf die Vorlesung vorbereiten konnte. | 2,79±1,01 | 3,71±1,10 | 0,0044* |
| 1. Ich bevorzuge Blended-Learning (Kombination unterschiedlicher Methoden/Medien: Präsenzunterricht und E-Learning) gegenüber dem alleinigen Präsenzunterricht. | 2,48±1,40 | 2,95±1,17 | 0,1981 |
| 1. Die curricular vorgegebene Anzahl von im Mittel 20 SWS/2 SWS ^c^ für die Vorlesung war zum Erlernen der theoretischen Kenntnisse angemessen. | 2,29±0,76 | 2,18±0,91 | 0,6689 |
| 1. Wie häufig (in %) haben Sie die Vorlesung besucht? | 1,00±0,00 | 1,36±0,58 | 0,0079* |
| 1. Wie bewerten Sie insgesamt die theoretische Wissensvermittlung im Rahmen der Vorlesung der technischen Propädeutik/Dentale Technologie ^e^? | 1,97±0,57 | 2,59±0,73 | 0,0020* |
| 1. **Im Praktikum** |  |  |  |
| Wie bewerten Sie die theoretische Wissensvermittlung, die im Rahmen des Praktikums erfolgte, hinsichtlich |  |  |  |
| 1. des Aufbaus? | 1,96±0,43 | 3,09±0,92 | 0,0000* |
| 1. der vorab zur Verfügung gestellten Lernziele zur jeweiligen Demo? | 2,41±0,84 | 2,57±0,68 | 0,4586 |
| 1. der Diskussionsmöglichkeiten? | 1,86±0,76 | 2,86±1,32 | 0,0032* |
| 1. der Art der Kommunikation der Dozentin/des Dozenten? | 1,68±0,77 | 2,73±1,08 | 0,0005* |
| 1. der Demos für das ganze Semester? | 2,57±0,96 | 2,91±1,02 | 0,2393 |
| 1. **^f^** |  |  |  |
| 1. **^f^** |  |  |  |
| Folgende Bestandteile waren, soweit angeboten, hilfreich für meinen Wissenserwerb: |  |  |  |
| 1. Videos | 2,00±1,04 | 2,32±0,72 | 0,2010 |
| 1. Kursunterlagen (z. B. Modelle, Präparate und Arbeitsblätter) | 1,66±0,67 | 2,18±0,66 | 0,0076* |
| 1. PDF-Dateien der Demos | 1,59±0,78 | 2,10±0,62 | 0,0137* |
| 1. Sonstige PDF-Dateien (Bewertungskriterien Modellation, Präparation etc.) | 1,48±0,69 | 1,77±0,81 | 0,1853 |
| 1. Hausaufgaben | 2,84±1,21 | 2,91±0,97 | 0,8581 |
| 1. Wie bewerten Sie insgesamt die theoretische Wissensvermittlung im Rahmen des Praktikums der technischen Propädeutik/Dentalen Technologie ^e^? | 2,07±0,59 | 2,86±0,83 | 0,0005* |
| **III. Praktische Wissensvermittlung** |  |  |  |
| 1. Die curricular vorgegebene Anzahl von im Mittel 20 SWS/3 SWS ^e^ für das Praktikum empfand ich zum Erlernen der praktischen Fertigkeiten als angemessen. | 2,00±0,96 | 4,14±0,79 | 0,0000* |
| 1. Die Frequenz der Praktikumstage empfand ich als angemessen. | 1,96±1,14 | 3,67±1,15 | 0,0000* |
| 1. Es befanden sich ausreichend viele ZahnärztInnen als BetreuerInnen im Kurs. | 1,38±0,68 | 3,36±1,05 | 0,0000* |
| 1. Es befanden sich ausreichend viele ZahntechnikerInnen als BetreuerInnen im Kurs. | 1,86±1,06 | 3,32±1,09 | 0,0000* |
| 1. Es befanden sich ausreichend viele studentische TutorInnen als BetreuerInnen im Kurs. | 1,97±0,94 | 1,68±0,72 | 0,2283 |
| Welche der folgenden Themen des Praktikums sind zu kurz gekommen? ^d^ |  |  |  |
| 1. Knetübungen | 1,97±1,05 | 2,50±1,50 | 0,1632 |
| 1. Gipsübungen | 1,69±0,71 | 2,50±1,30 | 0,0130* |
| 1. Aufwachsübungen | 2,14±1,13 | 2,86±1,49 | 0,0639 |
| 1. Situationsabformung, Modellherstellung | 2,17±1,04 | 3,14±1,39 | 0,0097* |
| 1. Übertragungsbogen, Einbau der Modelle und Splitcastkontrolle | 2,07±1,03 | 3,09±1,23 | 0,0031* |
| 1. Präparationen | 1,72±1,07 | 3,73±1,42 | 0,0000* |
| 1. Digitale Abformung und Design der Restauration | 2,50±1,32 | 3,09±1,34 | 0,1265 |
| 1. Die Anzahl der zu erbringenden praktischen Testate vor Ort (n=32) war in der curricular vorgegebenen Zeit gut umsetzbar. | 1,62±0,82 | 3,23±1,27 | 0,0000* |
| 1. Die Anzahl der intraoral simulierten Übungen (Präparationsübungen, Alginatabformungen etc.) empfand ich als angemessen. | 1,83±0,85 | 3,36±1,22 | 0,0000* |
| 1. Die Anzahl der extraoral simulierten Übungen (digitale Abformungen etc.) empfand ich als angemessen. | 2,14±0,80 | 2,64±1,22 | 0,1097 |
| 1. Die Anzahl der zahntechnisch simulierten Übungen (Gipsübungen, Aufwachsübungen etc.) empfand ich als angemessen. | 2,21±1,01 | 2,95±1,25 | 0,0275* |
| 1. Die Anzahl der Inseldienste empfand ich als angemessen. | 2,42±1,00 | 1,79±1,23 | 0,1309 |
| 1. ^f^ |  |  |  |
| 1. Die Praktikumszeit im Simulationslabor war ausreichend. | 1,62±0,94 | 4,50±0,60 | 0,0000* |
| 1. Die Praktikumszeit im zahntechnischen Labor war ausreichend. | 1,69±0,71 | 3,91±1,11 | 0,0000* |
| 1. Ich kann nach diesem Kurs einen Zahn nach den definierten Parametern zur Aufnahme eine Krone präparieren. | 1,69±0,76 | 2,55±1,06 | 0,0027* |
| 1. Ich kann nach diesem Kurs eine Krone nach den definierten Parametern modellieren. | 2,42±1,14 | 2,64±1,18 | 0,5285 |
| 1. Wie bewerten Sie insgesamt die praktische Wissensvermittlung im Rahmen des Praktikums der technischen Propädeutik/Dentalen Technologie ^e^? | 2,00±0,89 | 3,09±1,06 | 0,0004* |
| **IV. Sonstiges** |  |  |  |
| 1. Sind Ihnen die semesterbezogenen Lernziele dieser Lehrveranstaltung klar geworden? | 1,79±0,56 | 2,18±0,80 | 0,0583 |
| 1. Halten Sie den Anatomiekurs sinnvoll für den Einstieg in die technische Propädeutik/Dentale Technologie ^e^? | 2,28±1,16 | 1,45±0,67 | 0,0027* |
| 1. Haben Sie das Gefühl, dass Sie als Studierende/r von einer früheren beruflichen Erfahrung mit zahnärztlichen Fähigkeiten profitiert haben/hätten? | 1,35±0,57 | 1,72±1,18 | 0,2280 |
| 1. Haben Sie das Gefühl, dass Sie im späteren Studienverlauf von den Übungen im präklinischen Propädeutikkurs profitieren werden? | 1,85±0,82 | 1,86±0,85 | 0,9828 |
| 1. Welche Note würden Sie der Lehrveranstaltung insgesamt (Vorlesung + Praktikum) geben? | 2,07±0,80 | 2,86±0,73 | 0,0007* |
| 1. Wie bewerten Sie Ihren Wissenszuwachs nach dieser Lehrveranstaltung? | 1,72±0,59 | 2,14±0,73 | 0,0364* |
| **V. Lernziele** |  |  |  |
| 1. **der Vorlesung** |  |  |  |
| **Ich…** |  |  |  |
| 1. kann die Lehr-Lern-Situation in der angebotenen Veranstaltung reflektieren und evaluieren. | 2,04±0,58 | 2,24±0,90 | 0,4227 |
| 1. kann zahntechnische Arbeitsabläufe nachvollziehen. | 1,90±0,49 | 2,33±0,97 | 0,0676 |
| 1. kenne die Grundlagen für eine sichere Arbeitsumgebung sowie die Bedeutung der eigenen Gesundheit in Bezug zu beruflichen Gefahren und ihren Einfluss auf die Fähigkeit, den Arztberuf auszuüben. ^g^ | 1,62±0,56 | 1,95±0,84 | 0,1175 |
| 1. kann die verschiedenen Formen von Unterkieferbewegungen benennen und ihre Übertragung in den Artikulator anhand statischer und dynamischer Parameter erläutern. ^g^ | 2,72±1,03 | 3,05±1,17 | 0,3139 |
| 1. kann unterschiedliche Artikulatortypen unterscheiden, ihre Funktion erklären. ^g^ | 2,62±1,08 | 2,50±1,19 | 0,7104 |
| 1. kann die Stellung der Zähne im Ober– und Unterkiefer (sagittale und transversale Kompensationskurve) sowie die Breitenrelationen der Zähne erklären. ^g^ | 2,66±0,97 | 2,52±1,08 | 0,6603 |
| 1. kann die Morphologie der Okklusalfläche (tragende Höcker, Scherhöcker, Fissurenbild) in Abhängigkeit des jeweiligen Zahntyps erläutern. ^g^ | 1,62±0,49 | 2,05±0,90 | 0,0545 |
| 1. kann die gemeinsamen Zahnmerkmale erläutern. ^g^ | 1,72±0,59 | 1,68±0,72 | 0,8231 |
| 1. kenne die biologischen, chemischen, physikalischen, mechanisch-technologischen und biomechanischen Grundbegriffe und Definitionen der Werkstoffkunde. ^g^ | 3,38±1,12 | 3,55±1,18 | 0,6135 |
| 1. kann die physikalischen Grundlagen der Interaktion des menschlichen Körpers mit Werkstoffen und Biomaterialien ausführen und Eigenschaften wie Härte, Festigkeit und andere spezifische Materialeigenschaften charakterisieren sowie grundlegende Prinzipien präklinischer Prüfverfahren erläutern. | 3,17±0,93 | 3,59±1,10 | 0,1576 |
| 1. kann die biomechanischen Grundlagen und Folgen von einwirkenden Kräften auf unterschiedliche Werkstoffe/Restaurationen beschreiben und den physiologischen Belastungen in der Mundhöhle zuordnen. | 2,86±0,92 | 3,32±1,13 | 0,1296 |
| 1. kenne die erforderliche Präzision und Qualität für Arbeitsschritte und Restaurationen. ^g^ | 1,79±0,62 | 2,14±0,83 | 0,1134 |
| 1. kann die anwendungsbezogene Bedeutung der Oberflächenqualität (einer Restauration, eines Implantats) für die Langlebigkeit oder Einheilung charakterisieren. | 2,50±1,07 | 2,18±0,91 | 0,2615 |
| 1. kann Kriterien für die Beurteilung der erforderlichen Präzision von Restaurationen aufzählen und deren notwendige Einhaltung begründen. | 2,24±0,95 | 2,00±0,77 | 0,3284 |
| 1. kann Verfahren zur Prüfung der Präzision und Qualität beschreiben und Beispiele für deren Einsatz begründen. | 2,55±0,74 | 2,50±1,15 | 0,8600 |
| 1. kenne dentale Kunststoffe, metallische, keramische und biofunktionalisierte Werkstoffe. | 2,62±1,08 | 2,65±1,09 | 0,9265 |
| 1. kann Metalle und Legierungen aufzählen, sie toxikologisch einordnen, die Legierungsbildung beschreiben und die Werkstoffe in die jeweiligen Klassifizierungen einordnen. | 3,48±0,91 | 3,41±1,05 | 0,7947 |
| 1. kann kieferorthopädische Drahtligaturen, deren Zusammensetzung, sowie indikationsbezogene Eignung erklären. | 4,04±1,17 | 2,43±0,98 | 0,0000* |
| 1. kann Spezifika der Reaktionsweise und Verarbeitung dentaler Kunststoffe in Hinblick auf die indikationsgerechte Verwendung in den unterschiedlichen Fachgebieten beschreiben. | 3,42±1,10 | 3,21±1,03 | 0,5110 |
| 1. kann Methoden zur Biofunktionalisierung von Materialien vergleichen und Beispiele beschreiben. | 3,26±0,86 | 3,14±1,11 | 0,6934 |
| 1. kann die angemessenen Substanzen und Techniken für die Desinfektion von Materialien und Restaurationen vor dem Transport unter Berücksichtigung der Eignung und Interaktion mit Biomaterialien und Werkstoffen auswählen. ^g^ | 2,74±1,26 | 2,67±0,97 | 0,8186 |
| 1. kann die Anforderungen an Reinigungsfähigkeit, Sterilisierbarkeit, Desinfektion und Materialeigenschaften für zahnärztliche Instrumente und Hilfsmittel beschreiben und anwendungsspezifische Maßnahmen veranlassen sowie geeignete Substanzen identifizieren. | 2,45±1,02 | 2,18±1,22 | 0,4126 |
| 1. kann werkstoffbezogene Methoden zur Diagnose und Differenzialdiagnose und kann therapie- und indikationsbezogen die geeigneten Materialien unter Berücksichtigung von Materialunverträglichkeiten auswählen. | 3,00±0,88 | 2,95±0,92 | 0,8569 |
| 1. kann im Rahmen restaurativer Maßnahmen geeignete Materialien nutzen und Verfahren zur Erfassung und Übertragung der klinischen Situation beschreiben (intraorale Digitalisierung, digitaler Workflow). | 2,78±0,97 | 2,62±1,07 | 0,5992 |
| 1. kenne im Rahmen restaurativer Maßnahmen die Übertragung und Archivierung der klinischen Situation (analoge bzw. digitale Werkzeuge und Technologien für die Herstellung von Zahnersatz). | 2,64±0,83 | 2,73±1,32 | 0,7942 |
| 1. kann im Rahmen restaurativer Maßnahmen das technisch/technologische Vorgehen bei der Restaurationsfertigung und die notwendigen Werk- und Hilfsstoffe sowie Geräte für die einzelnen Arbeitsschritte beschreiben. ^g^ | 2,56±1,01 | 2,67±1,06 | 0,7159 |
| 1. kann indikationsbezogen Materialien für implantologische Maßnahmen benennen. | 3,42±1,21 | 2,14±0,73 | 0,0001* |
| 1. kann im Rahmen der kieferorthopädischen Therapie indikationsbezogen Materialien für die Therapie mit festsitzenden und herausnehmbaren Apparaturen benennen und hinsichtlich ihrer Eignung bewerten. | 3,54±1,25 | 2,32±0,99 | 0,0006* |
| 1. kann die zahnärztliche Röntgenbildgebung erläutern. | 3,15±1,46 | 2,32±1,17 | 0,0321* |
| 1. kann die zahnärztliche Röntgenbildgebung im Kontext der Zahnansprache erläutern. | 2,96±1,40 | 2,38±1,07 | 0,1096 |
| 1. kann CAD/CAM ^h^-Prinzipien der indirekten Restauration erläutern. | 3,21±1,10 | 3,14±1,21 | 0,8149 |
| 1. kann Indikationen und Kontraindikationen zur zahnärztlichen Implantation darstellen. | 3,08±1,44 | 1,55±0,60 | 0,0000* |
| 1. kann die wesentlichen Konstruktionsmerkmale gängiger Zahnimplantattypen benennen. | 3,64±1,25 | 2,18±0,96 | 0,0000* |
| 1. kann Verfahren zur präimplantären Diagnostik beschreiben. | 3,32±1,13 | 2,24±0,70 | 0,0006* |
| 1. kann Maßnahmen und Schritte bei der Therapie des Zahnverlustes für verschiedene Formen des Zahnersatzes erläutern. | 2,63±1,08 | 1,86±0,56 | 0,0027* |
| 1. kann Zahnpräparationen substanzschonend und unter Berücksichtigung der Anforderungen an Präparationsform und Platzbedarf indikationsgerecht unterscheiden. ^g^ | 1,86±0,69 | 1,73±0,63 | 0,4726 |
| 1. kann Abformungen mit dem Schwerpunkt der Reproduktion von Zähnen bzw. Präparationen unter Berücksichtigung von Indikation und Materialeigenschaften erklären. | 2,10±0,77 | 2,21±0,92 | 0,6768 |
| 1. kann den Ablauf von Abformungen mit dem Schwerpunkt der Reproduktion von zahnlosen Kiefern und Kieferabschnitten unter Berücksichtigung von Indikation und Materialeigenschaften erklären. ^g^ | 2,77±1,11 | 2,62±1,16 | 0,6549 |
| 1. kann grundlegende zahntechnische Arbeitsschritte zur Herstellung von Modellen und Übertragung der Patientensituation erklären und zur Qualitätskontrolle berücksichtigen. ^g^ | 2,38±0,96 | 2,19±1,12 | 0,7796 |
| 1. kann festsitzende Versorgungen bei unterschiedlichen Befunden indikationsgerecht beschreiben. ^g^ | 2,88±1,14 | 2,55±0,86 | 0,2472 |
| 1. kann unterschiedliche Formen implantatgetragener Versorgungen indikationsgerecht nennen. | 3,20±1,08 | 2,14±0,71 | 0,0002* |
| 1. kann Maßnahmen zur Wiederherstellung der Funktion und Ästhetik von bestehenden Versorgungen indikationsgerecht beschreiben. | 2,77±1,03 | 2,23±0,87 | 0,0543 |
| 1. **des Praktikums** |  |  |  |
| **Ich…** |  |  |  |
| 1. kann mit eigenen Fehlern und den Fehlern anderer angemessen umgehen und beherrsche eine situationsangemessene Fehlerkommunikation. | 1,69±0,60 | 2,09±1,15 | 0,1475 |
| 1. kann unterschiedliche Artikulatortypen unterscheiden und bedienen. | 2,61±1,03 | 2,75±1,16 | 0,6629 |
| 1. kann die Morphologie der Okklusalfäche (tragende Höcker, Scherhöcker, Frissurenbild) in Abhängigkeit des jeweiligen Zahntyps praktisch (Gips, Wachs, Knete, Komposit etc.) reproduzieren. ^i^ | 1,83±0,66 | 2,59±1,14 | 0,0086* |
| 1. kann die gemeinsamen Zahnmerkmale in meiner Tätigkeit berücksichtigen. | 1,62±0,65 | 2,23±0,92 | 0,0358* |
| 1. führe Maßnahmen und Schritte bei der Therapie des Zahnverlustes für verschiedene Formen des Zahnersatzes durch. | 2,48±1,12 | 2,68±1,00 | 0,5288 |
| 1. kann Zahnpräparationen substanzschonend und unter Berücksichtigung der Anforderungen an Präparationsform und Platzbedarf indikationsgerecht durchführen (z. B. Black Klasse I, Kronen Front- und Seitenzahnbereich). ^i^ | 2,18±0,90 | 2,41±0,91 | 0,3769 |
| 1. kann Abformungen mit dem Schwerpunkt der Reproduktion von Zähnen unter Berücksichtigung von Indikationen und Materialeigenschaften durchführen. | 2,22±0,64 | 2,86±0,94 | 0,0099* |
| 1. kann Abformungen mit dem Schwerpunkt der Reproduktion von zahnlosen Kiefern und Kieferabschnitten unter Berücksichtigung von Indikationen und Materialeigenschaften durchführen. | 2,96±1,11 | 3,84±1,12 | 0,0127* |
| 1. kann grundlegende zahntechnische Arbeitsschritte zur Herstellung von Modellen und Übertragung der Patientensituation durchführen und zur Qualitätskontrolle nutzen. | 2,10±0,56 | 2,62±1,07 | 0,0534 |
| 1. kann festsitzende Versorgungen bei unterschiedlichen Befunden indikationsgerecht zuordnen. | 2,68±1,06 | 2,30±0,86 | 0,1798 |

^a^1 = sehr gut/stimme voll zu – 5 = mangelhaft/stimme gar nicht zu

^b^ TPK = Kurs der Technischen Propädeutik

^c^ ZPDT = Zahnmedizinische Propädeutik mit Schwerpunkt Dentale Technologie

^d^ Aufgrund der negativen Formulierung der Fragen 8-22 und 46-52 wurde eine Umpolung ihrer Ergebnisse vorgenommen, um die Kongruenz in der Aussagekraft mit den übrigen Fragen des Fragebogens zu gewährleisten.

^e^ An dieser Stelle differierte die Frage im Fragebogen an den TPK (links) bzw. an den ZPDT (rechts): (TPK/ZPDT).

^f^ Fragen 33, 34 und 58 wurden lediglich für die Lehrveranstaltung ZPDT abgefragt.

^g^ Lernziele, die im Rahmen der Selbst- und Fremdeinschätzung den entsprechenden Klausurfragen zugeordnet wurden.

^h^ CAD/CAM = Computer-Aided Design/Computer-Aided Manufacturing

^i^ Lernziele, die im Rahmen der Selbst- und Fremdeinschätzung den entsprechenden praktischen Prüfungsaufgaben zugeordnet wurden.

*Statistische Signifikanz (p ≤ 0,05)

**Tabelle 4 – Ergebnisse der Evaluation zur Erfassung spezieller Lehr- und Lernbedingungen aus Sicht der Lehrenden (Parameter Nr. 4 ^a^)**

|  | **TPK ^b^** | **ZPDT ^c^** | **Signifikanz** |
| --- | --- | --- | --- |
| **I. Theoretische Wissensvermittlung** |  |  |  |
| - 1. **in der Vorlesung** |  |  |  |
| Wie bewerten Sie die theoretische Wissensvermittlung, die im Rahmen der Vorlesung erfolgte, hinsichtlich? |  |  |  |
| 1. des Aufbaus? | 2,12±0,64 | 1,83±0,41 | 0,3206 |
| 1. der vorab (in der Vorlesung, auf der Homepage) zur Verfügung gestellten Lernziele zur jeweiligen Vorlesungseinheit? | 2,57±0,98 | 2,75±1,04 | 0,7366 |
| 1. der Diskussionsmöglichkeit mit den Studierenden? | 2,22±1,09 | 2,00±0,93 | 0,6565 |
| 1. der Art der Kommunikation mit den Studierenden? | 1,67±0,71 | 1,62±0,52 | 0,8909 |
| 1. der PowerPoint-Präsentationen? | 2,11±0,78 | 1,75±0,71 | 0,3332 |
| 1. der hochgeladenen PDF-Dateien der Vorlesungsfolien? | 1,89±0,78 | 1,62±0,74 | 0,4870 |
| 1. der Literaturempfehlungen? | 2,33±1,12 | 2,00±0,76 | 0,4792 |
| Welche der folgenden Themen der Vorlesungsreihe sind zu kurz gekommen? ^d^ |  |  |  |
| 1. Orale Strukturen (z. B. Nomenklatur und Topografie des Kauorgans, Zahnansprache, Morphologie der Zähne und okklusales Relief, Zahnentwicklung) | 3,75±1,49 | 4,00±0,89 | 0,7033 |
| 1. Grundlagen der Okklusion | 4,14±1,46 | 3,86±1,07 | 0,6847 |
| 1. Hygiene und Arbeitssicherheit (inkl. Ergonomie am Arbeitsplatz) | 3,25±1,58 | 3,71±1,25 | 0,5374 |
| 1. Unterkieferbewegungen, Artikulatoren (z. B. Übersicht Artikulatoren, Winkel Einstellungen, Übertragungsbogen, Einbau der Modelle im Artikulator, Kaumuskulatur und Kiefergelenk) | 3,57±1,62 | 3,86±1,21 | 0,7158 |
| 1. Situationsabformung und Modellherstellung – konventionell und digital | 3,57±1,62 | 3,57±1,40 | 1,0000 |
| 1. Präparationen (Vollkrone Front- und Seitenzahnbereich) | 3,43±1,81 | 4,00±1,20 | 0,4937 |
| 1. Werkstoffkunde I (z. B. Basics, Gipse und Wachse) | 3,89±1,27 | 3,71±1,38 | 0,7991 |
| 1. Werkstoffkunde II (z. B. Kennzahlen/Prüfverfahren, Metalle) | 3,71±1,25 | 3,71±1,38 | 1,0000 |
| 1. Werkstoffkunde III (z. B. Biokompatibilität und Unverträglichkeit) | 3,57±1,40 | 3,86±1,21 | 0,6904 |
| 1. Arten von Zahnersatz, Qualitätskriterien | 3,43±1,40 | 3,71±1,11 | 0,6800 |
| 1. Dentale Technologie in der Kieferorthopädie | 3,00±1,73 | 4,00±1,00 | 0,4465 |
| 1. Parodontale Röntgendiagnostik | 2,50±2,12 | 3,50±0,71 | 0,6248 |
| 1. Implantologie | 2,50±2,12 | 3,50±0,71 | 0,6248 |
| 1. Implantologie aus parodontologischer Sicht | 2,50±2,12 | 4,00±0,00 | 0,5000 |
| 1. Funktion und Ästhetik | 3,40±1,52 | 4,29±0,76 | 0,2786 |
| 1. Die Unterrichtsmaterialien wurden rechtzeitig zur Verfügung gestellt, sodass sich die Studierenden gut auf die Vorlesung vorbereiten konnten. | 1,78±1,09 | 1,88±1,13 | 0,8595 |
| 1. Die curricular vorgegebene Anzahl von im Mittel 20 SWS/2 SWS^e^ (Semesterwochenstunden) für die Vorlesung war zum Erlernen der theoretischen Kenntnisse angemessen. | 2,00±1,00 | 3,33±1,51 | 0,0932 |
| 1. Wie bewerten Sie insgesamt die theoretische Wissensvermittlung im Rahmen der Vorlesung der technischen Propädeutik/Dentalen Technologie^e^? | 1,67±0,50 | 2,00±1,00 | 0,4421 |
| 1. **Im Praktikum** |  |  |  |
| Wie bewerten Sie die theoretische Wissensvermittlung, die im Rahmen des Praktikums erfolgte, hinsichtlich |  |  |  |
| 1. des Aufbaus? | 2,12±0,83 | 2,29±1,11 | 0,7602 |
| 1. der vorab zur Verfügung gestellten Lernziele zur jeweiligen Demo? | 3,43±0,79 | 1,62±0,52 | 0,0004* |
| 1. der Diskussionsmöglichkeiten? | 2,50±1,20 | 2,43±1,27 | 0,9129 |
| 1. der Art der Kommunikation mit den Studierenden? | 1,88±0,83 | 1,71±0,49 | 0,6528 |
| 1. der Demos für das ganze Semester? | 2,43±1,27 | 1,83±0,75 | 0,3217 |
| 1. Wie bewerten Sie insgesamt die theoretische Wissensvermittlung im Rahmen des Praktikums der technischen Propädeutik/Dentalen Technologie ^e^? | 2,25±0,89 | 2,00±1,22 | 0,7044 |
| **II. Praktische Wissensvermittlung** |  |  |  |
| 1. Die curricular vorgegebene Anzahl von im Mittel 20 SWS/3drei SWS^e^ (Semesterwochenstunden) für das Praktikum war zum Erlernen der praktischen Fertigkeiten angemessen. | 1,43±0,53 | 4,17±0,98 | 0,0004* |
| 1. Die Frequenz der Praktikumstage war angemessen. | 1,29±0,76 | 3,67±1,51 | 0,0096* |
| 1. Es befanden sich ausreichend viele ZahnärztInnen als BetreuerInnen im Kurs. | 2,00±0,82 | 2,43±1,27 | 0,4702 |
| 1. Es befanden sich ausreichend viele ZahntechnikerInnen als BetreuerInnen im Kurs. | 2,43±0,98 | 3,00±1,22 | 0,4139 |
| 1. Es befanden sich ausreichend viele studentische TutorInnen im Kurs. | 2,71±0,95 | 2,67±1,51 | 0,9483 |
| Welche der folgenden Themen des Praktikums sind zu kurz gekommen? ^d^ |  |  |  |
| 1. Knetübungen | 4,71±0,49 | 4,33±0,82 | 0,3469 |
| 1. Gipsübungen | 4,43±0,79 | 4,00±0,63 | 0,2998 |
| 1. Aufwachsübungen | 4,29±0,76 | 3,67±1,03 | 0,2549 |
| 1. Situationsabformung, Modellherstellung | 4,00±1,00 | 2,67±1,51 | 0,0997 |
| 1. Übertragungsbogen, Einbau der Modelle und Splitcastkontrolle | 4,14±0,90 | 3,00±0,63 | 0,0221* |
| 1. Präparationen | 4,29±1,11 | 3,67±1,51 | 0,4271 |
| 1. Digitale Abformung und Design der Restauration | 2,20±1,30 | 3,83±1,17 | 0,0612 |
| 1. Die Anzahl der zu erbringenden praktischen Testate vor Ort (n=32) war in der curricular vorgegebenen Zeit gut umsetzbar. | 1,29±0,49 | 4,00±1,00 | 0,0020* |
| 1. Die Anzahl der intraoral simulierten Übungen (Präparationsübungen, Alginatabformungen etc.) war angemessen. | 2,00±1,53 | 2,80±1,79 | 0,4414 |
| 1. Die Anzahl der extraoral simulierten Übungen (digitale Abformungen etc.) war angemessen. | 2,60±1,52 | 2,60±1,67 | 1,0000 |
| 1. Die Anzahl der zahntechnisch simulierten Übungen (Gipsübungen, Aufwachsübungen etc.) war angemessen. | 1,71±0,76 | 2,25±1,50 | 0,5419 |
| 1. Die Praktikumszeit im Simulationslabor war ausreichend. | 1,29±0,49 | 4,40±0,89 | 0,0005* |
| 1. Die Praktikumszeit im zahntechnischen Labor war ausreichend. | 1,43±0,53 | 4,00±0,82 | 0,0033* |
| 1. Wie bewerten Sie insgesamt die praktische Wissensvermittlung im Rahmen des Praktikums der technischen Propädeutik/Dentalen Technologie^e^? | 2,00±0,89 | 2,60±0,89 | 0,2978 |
| **III. Sonstiges** |  |  |  |
| 1. Welche Note würden Sie der Lehrveranstaltung insgesamt (Vorlesung + Praktikum) geben? | 1,83±0,75 | 2,43±0,79 | 0,1919 |

^a^1 = sehr gut/stimme voll zu – 5 = mangelhaft/stimme gar nicht zu

^b^ TPK = Kurs der Technischen Propädeutik

^c^ ZPDT = Zahnmedizinische Propädeutik mit Schwerpunkt Dentale Technologie

^d^ Aufgrund der negativen Formulierung der Fragen 8-22 und 37-43 wurde eine Umpolung ihrer Ergebnisse vorgenommen, um die Kongruenz in der Aussagekraft mit den übrigen Fragen des Fragebogens zu gewährleisten.

^e^ An dieser Stelle differierte die Frage im Fragebogen an den TPK (links) bzw. an den ZPDT (rechts): (TPK/ZPDT).

*Statistische Signifikanz (p ≤ 0,05)
